# The Effect of Sex on the Therapeutic Efficiency of Immune Checkpoint Inhibitors: A Systematic Review and Meta-Analysis Based on Randomized Controlled Trials

**DOI:** 10.3390/cancers16020382

**Published:** 2024-01-16

**Authors:** Xingyu Zhong, Jianxuan Sun, Na Zeng, Yifan Xiong, Ye An, Shaogang Wang, Qidong Xia

**Affiliations:** Department and Institute of Urology, Tongji Hospital, Tongji Medical College, Huazhong University of Science and Technology, No.1095 Jiefang Avenue, Wuhan 430030, China; xingyuzhong00@126.com (X.Z.); sunjianxuan123@126.com (J.S.); zeng_hei@163.com (N.Z.); u201910348@hust.edu.cn (Y.X.); ay121253@163.com (Y.A.)

**Keywords:** sex, immunotherapy, immune checkpoint inhibitors, randomized controlled study, meta-analysis

## Abstract

**Simple Summary:**

Sex is an important variable that can affect immunity, both in the cellular and humoral sense. This may result from differences in genes, hormones, the environment, and commensal microbiome composition. Although some researchers have investigated the relationship between the efficacy of immunotherapy and sex, their conclusions are contradictory. Based on immune checkpoint inhibitors (ICI) in cancer, we conducted an updated systematic review and meta-analysis to explore whether sex has an impact on the efficacy of immunotherapy. A total of 103 articles of randomized controlled trials were retrieved from the bibliographic database. The results show that both male and female patients treated with ICI showed a lower risk of death than control subjects, with females achieving smaller gains (*p* = 0.02). And in the PD-1 subgroup, male patients showed a better response to ICI (*p* = 0.0073). We found that sex is a factor affecting the efficacy of ICI, and that males were more likely to benefit from ICI treatment.

**Abstract:**

Background: Sex is an important factor influencing the immune system, and the distribution of tumors, including their types and subtypes, is characterized by sexual dichotomy. The aim of this study was to investigate whether there is an association between sex and the treatment effect of immune checkpoint inhibitors (ICI). Methods: Four bibliographic databases were searched. Studies of randomized controlled trials (RCTs) assessing the efficacy of ICI were identified and used, and the primary endpoint was the difference in efficacy of ICI between males and females, presented as overall survival (OS), progression-free survival (PFS) and recurrence-free survival (RFS). The study calculated the pooled HRs and 95% CIs for OS, PFS and RFS for males and females using a random effects model or a fixed effects model, and thereby assessed the effect of sex on the efficacy of ICI treatment. This study is registered with PROSPERO (CRD42022370939). Results: A total of 103 articles, including a total of 63,755 patients with cancer, were retrieved from the bibliographic database, of which approximately 70% were males. In studies with OS as the outcome, the combined hazard ratio (HR) was 0.77 (95% CI 0.74–0.79) for male patients treated with ICI and 0.81 (95% CI 0.78–0.85) for female patients compared to controls, respectively. The difference in efficacy between males and females was significant. Conclusions: ICI therapy, under suitable conditions for its use, has a positive impact on survival in various types of tumors, and male patients benefit more than females. It may be necessary to develop different tumor immunotherapy strategies for patients of different sexes.

## 1. Introduction

Intersexual differences often manifest as differences in sex chromosomes and hormone levels between men and women, and this difference often leads to distinct immune responses between males and females. According to reports, females show stronger immune responses in most cases compared to males [1,2]. This distinction is evident in the observation that women are more susceptible to autoimmune diseases, while being less susceptible to infectious diseases [3,4]. Regarding cancer, males generally have a higher risk of developing cancer compared to females [5]. Additionally, certain cancer types, such as lung cancer, demonstrate a higher mortality rate among males. Various factors can contribute to this disparity, including distinct lifestyles between males and females. Moreover, it is crucial to acknowledge the differences in the immune systems of males and females as an influencing factor.

Immunotherapy, represented by immune checkpoint inhibitors (ICI), has introduced a novel approach to cancer treatment. ICI primarily encompass anti-PD-1 antibodies, anti-PD-L1 antibodies and anti-CTLA-4 antibodies. These agents function by impeding the PD-1-PD-L1 or the CTLA-4 signaling pathway between tumor cells and immune cells, thereby inhibiting immune evasion by tumors [6]. The fundamental objective of ICI therapy is to bolster the body’s immune response, resulting in reduced harm to normal tissues compared to conventional chemotherapy, as well as improved therapeutic efficacy in certain cases [7].

Despite the considerable advantages of ICI treatment in antitumor therapy, a majority of patients do not respond favorably to it [8]. Therefore, it is imperative to investigate the factors influencing the efficacy of ICI therapy. Additionally, considering that females generally exhibit superior immune strength compared to males in the absence of intervention, the impact of ICI therapy in enhancing antitumor immune responses may be relatively less pronounced in females than in males. This difference in response between males and females may contribute to variations in the efficacy of ICI therapy. However, previous studies have inadequately considered the influence of sex differences on ICI efficacy, and the limited availability of data regarding female patients may lead to erroneous conclusions, as most studies have been predominantly conducted on males. Fortunately, there has been a recent increase in the number of clinical trials investigating ICI therapies, which have provided supplementary data on relevant sex differences. Therefore, in this study, we conducted a systematic review and meta-analysis of randomized controlled clinical studies to evaluate the impact of sex on the efficacy of ICI therapy.

## 2. Materials and Methods

### 2.1. Selection Criteria

The inclusion and exclusion criteria were as follows: (1) the type of studies were phase II/III clinical trials and RCTs; (2) the subjects were treated with PD-1, PD-L1 or CTLA-4, or combinations of them; (3) the outcome of interest was HR for survival outcome, including overall survival (OS), progression-free survival (PFS) and recurrence-free survival (RFS); (4) the studies included only the most recent patient dataset reported in different articles; (5) studies without data analyzed according to sex were excluded; (6) studies with no full text or lacking valuable data, including numbers of patients (categorized by sex), treatment phase, treatment of controls, interventions and HR of survival outcomes, were excluded.

### 2.2. Search Strategy

**Figure 1:**
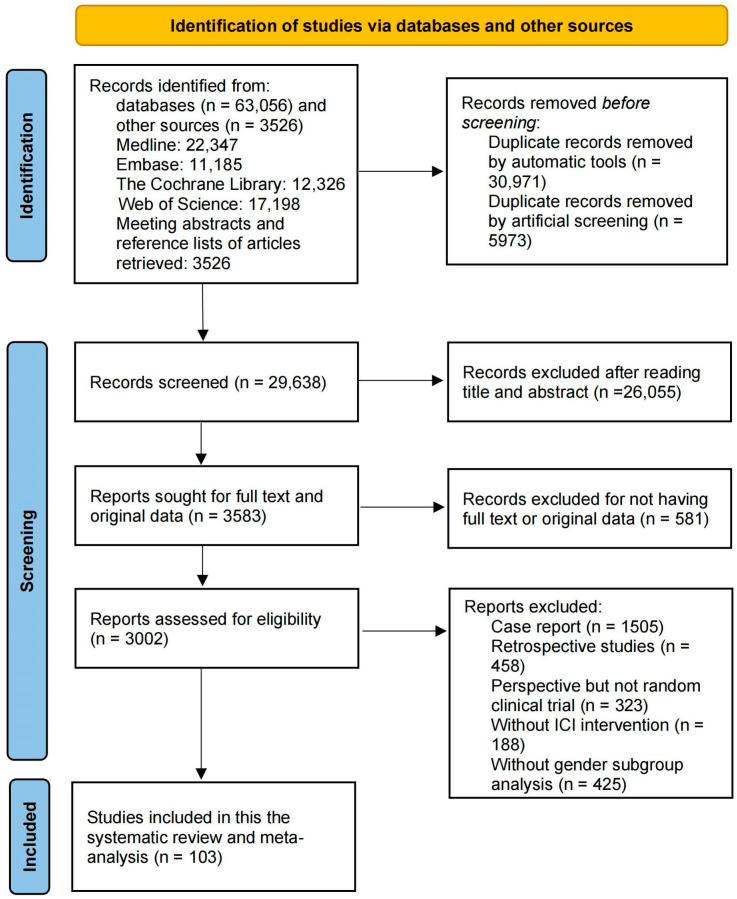
Study selection PRISMA (preferred reporting items for systematic reviews and meta-analyses) flowchart for study selection for the systematic review on the effects of ICI, with data analyzed according to male and female subgroups. ICI, immune checkpoint inhibitors.

This study was conducted according to the preferred reporting items for systematic reviews and meta-analyses (PRISMA) 2020 reporting guidelines (Supplementary Materials Table S1) [9]. Four bibliographic databases, namely PubMed (Medline), EMBASE, Cochrane Library and Web of Science, were searched for studies assessing the relationship between sex and ICI treatment outcomes, from initiation to 28 November 2023. Conference abstracts were also included. The databases were searched using keywords such as “Immune Checkpoint Inhibitors”, “PD-1 “, “PD-L1”, “CTLA-4”, “Neoplasms” and “Cancers”. Detailed search strategies in the different databases are described in Supplementary Materials Tables S2–S5. Two reviewers, Z.X. and S.J., searched abstracts during the screening procedure and selected them according to the search criteria. Differences regarding inclusion or exclusion were resolved by consensus by the third author (Z. N.), and the Endnote application (version X9) was used to remove duplicates and apply inclusion criteria. The PRISMA flowchart in Figure 1 was used to describe the literature search process. This systematic review and meta-analysis study is registered with PROSPERO (CRD42022370939).

### 2.3. Data Extraction

This research used a designed data extraction form to extract information from the included studies. The data extraction form consists of bibliographic information and background information. The bibliographic information includes the name of the author, year of publication, journal name and title. The background information contains the region of the study conducted, age, number of male/female patients and order of treatment (whether it was first-line or not). In addition, the background information also includes tumor type, type of ICI, and mean follow-up time. The detailed characteristics of the articles included in this study are shown in Table 1. The outcomes of interest were the survival outcomes of patients, including OS, PFS and RFS, males and females separately.

### 2.4. Literature Quality Assessment

All of the included studies used the modified Jadad scale [113] to evaluate the quality of trials in four dimensions (Supplementary Materials Table S6). A study with a total score of 1–3 was considered as low-quality literature, and 4–7 as high-quality literature. Two researchers, X. Y. F. and Z. N., performed the risk of bias and literature quality assessment, and discrepancies were resolved by consensus with the third researcher, A. Y.

### 2.5. Data Synthesis and Statistical Analysis

In this study, the effectiveness of ICI in patients with cancer was evaluated using a random effects (RE) model or a fixed effects (FE) model [114], on the basis of different survival outcome (OS, PFS and RFS) classifications. Then, the relationship between ICI and survival outcomes was further evaluated in patients of different sexes, and 95% CIs were calculated to assess the relationship between sex and ICI treatment effects. The heterogeneity between studies was also analyzed using the standard Cochrane chi-square χ2 (Cochrane’s Q) test with a significance level of α = 0.10, as well as the I2 test [115]. The I2 test describes the percentage of variation between studies, and an I2 statistic of ≥50% indicates considerable heterogeneity [116]. Subgroup analysis stratified by parameters such as tumor types and category of ICI was performed to identify potential sources of heterogeneity. The study also performed a meta-regression, using parameters such as age and follow-up duration, which could be potential confounding factors across the studies. Begg’s [117] and Egger’s [118] tests were used to identify publication bias. Using contour-enhanced funnel plots, other causes of publication bias were identified by examining the symmetry of the funnel plots. Finally, the study carried out sensitivity analysis and cumulative meta-analysis by incrementally adding or omitting included studies, and also applied trim-and-fill methods to assess the effect of publication bias [119]. This study used the “meta” package [120] and the “metagen” command [121] of R software version 4.2.0 for data processing and statistical analysis. All *p* values were two-sided, and *p* < 0.05 was considered a significant difference.

## 3. Results

This study retrieved 66,582 publications from electronic databases and other sources. However, after applying the inclusion and exclusion criteria (shown in Figure 1), 66,479 articles were excluded, and 103 articles were included in this systematic review and meta-analysis study. Of the 66,479 excluded articles, 30,971 duplicates were removed using the automated tool in the Endnote application, and 5973 were removed using manual identification by reviewers. Furthermore, after reading the titles and abstracts, 26,055 articles were removed, and 581 articles were excluded for lack of full text or raw data. After reading the full text, 1505 case reports, 458 retrospective studies and 323 non-RCT prospective studies were excluded. Ultimately, 188 articles were removed for not including ICI intervention, and another 425 articles were removed for lacking sex subgroup analyses.

### 3.1. Characteristics of Included Studies and Patients

**Figure 2:**
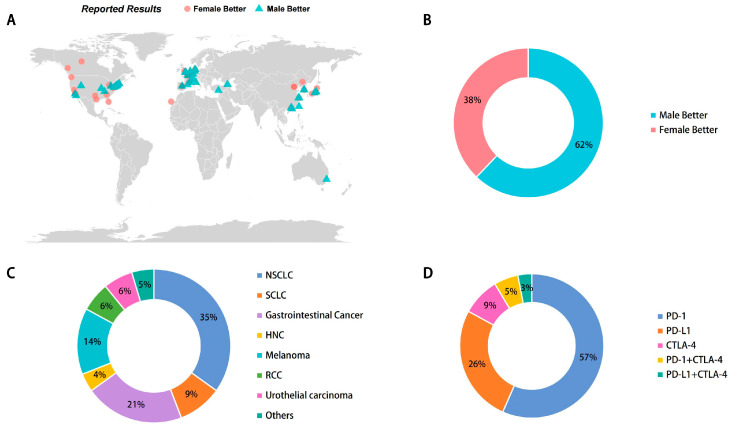
Characteristics of the studies. (**A**) Regional distribution of included studies and comparison of HR size between males and females (better for males represents a smaller HR for males and vice versa). (**B**) Male-to-female ratio of study subjects, with the number of males to females being approximately 1:1.6. (**C**,**D**) Proportion of study subjects by tumor type (**C**) and ICI drug type (**D**) to overall counts.

The characteristics of the 103 articles included in this study are shown in Table 1. All 103 articles were analyses of data from phase 2/3 clinical studies, and the outcomes of interest included OS, PFS and RFS in patients treated with ICI, which corresponded to 77, 33 and 12 articles, respectively. All but 21 studies were conducted in a single country, and the remaining studies involved multiple countries with a cumulative scope of Europe, North America, Asia, Africa and Oceania (Figure 2). There were 63,755 cancer patients, and the number of patients in each study ranged from 61 to 1764. There were 76 studies that included follow-up information, with an average follow-up time ranging from 5 months to 7 years (Table 1).

In these studies, the average age of the patients was over 60 years, and the proportion of male patients was approximately 70%. A review of the literature found that more than sixty percent of the studies revealed that males showed better outcomes after ICI treatment, but nearly forty percent still provided the contrary conclusion. Meanwhile, anti-PD-1 and anti-PD-L1 antibodies were the most commonly used ICI treatment, and lung cancer emerged as the most preferred ICI treatment candidate among all tumor types.

### 3.2. Quality Assessment of the Included Studies

The Jadad assessment criteria were used to assess the quality of RCT studies in this study, as shown in Supplementary Materials Table S6. All of the experiments were conducted via random assignment. The use of the Jadad criteria indicated that all of the articles included in this study were of high or moderate quality.

### 3.3. Effect of Sex on OS after ICI Treatment

Of all the trials with OS as an outcome, 41 used anti-PD-1 therapy, 20 used anti-PD-L1 therapy, 8 used anti-CTLA-4 antibodies and the other 10 used a combination of anti-PD-1/PD-L1 and anti-CTLA-4 therapy.

**Figure 3:**
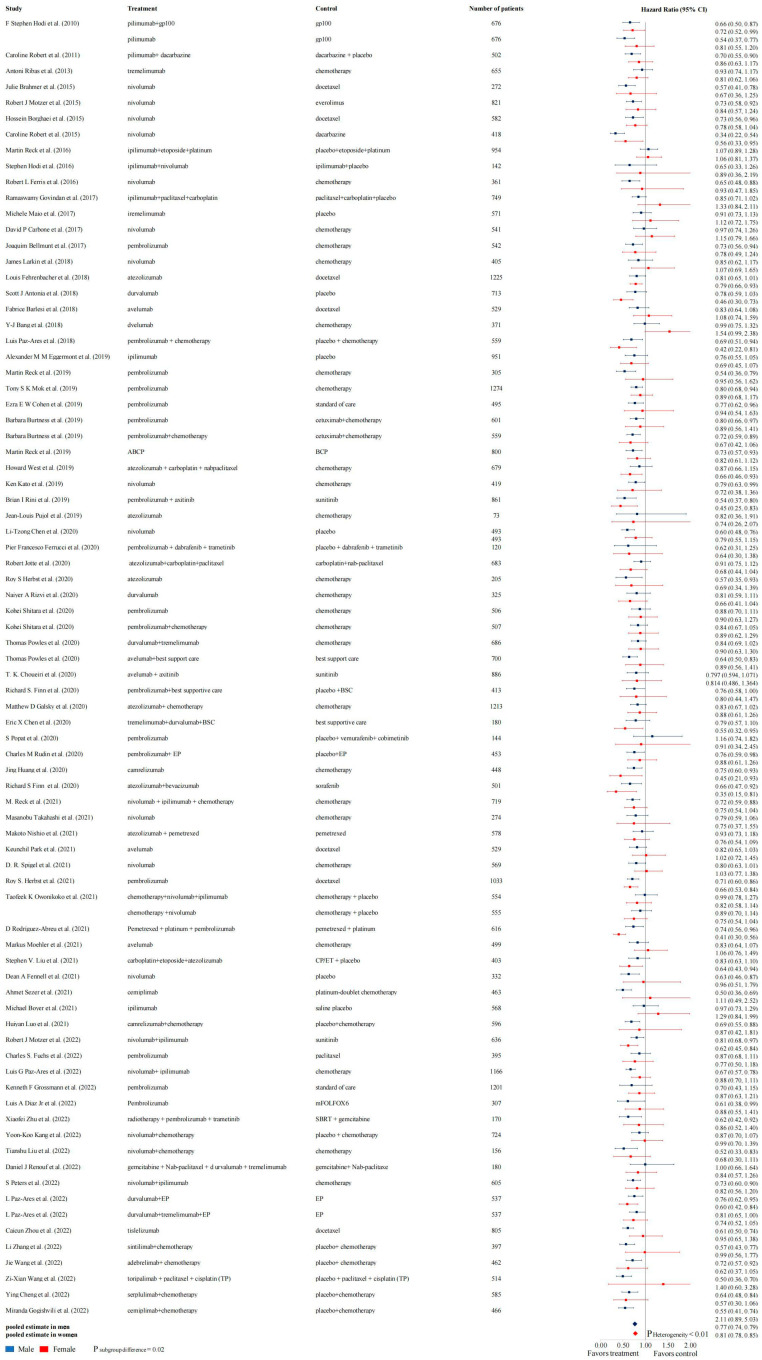
Hazard ratios for death in the intervention and control groups according to sex, with OS as the outcome. (The references cited in the figure are consistent with Table 1).

Among male patients, those treated with ICI showed a lower risk of death than those in the control group (pooled overall survival HR 0.77, 95% CI 0.74–0.79) (Figure 3). The cumulative meta-analysis in Figure S1A and sensitivity analysis in Figure S1B showed a relatively stable result. This study used Egger’s test to identify publication bias. Both Egger’s test (t = −3.16 and *p*-value = 0.0022) and Begg’s test (z = −3.06, *p*-value = 0.0022) results indicated significant publication bias.

Similarly, female patients treated with ICI also benefited, but with smaller gains compared to male patients (pooled overall survival HR 0.81, 95% CI 0.78–0.85) (Figure 3). The cumulative meta-analysis in Figure S2A and sensitivity analysis in Figure S2B showed a relatively stable result. This study used Egger’s test to identify publication bias. Both Egger’s test (t = −0.37, *p*-value = 0.716) and Begg’s test (z = −1.07, *p*-value = 0.283) results indicated no significant publication bias.

Overall, a certain amount of heterogeneity existed between studies (I^2^ = 35%, *p* < 0.01). Comparing the differences between men and women on the effect of ICI treatment in terms of improvement in OS was more pronounced in male patients with ICI treatment (*p* = 0.02). An interactional HR analysis of men and women showed that the ICI intervention group exhibited a lower risk of death than the control group (HR 0.78, 95% CI 0.76–0.80) (Figure S3).

**Figure 4:**
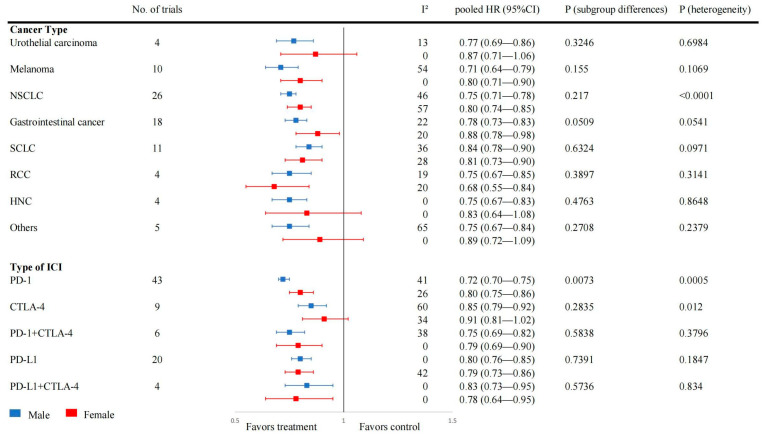
Subgroup analysis of hazard ratios for death in tumor patients stratified by tumor type and ICI drug type, by sex, with OS as the outcome.

A subgroup analysis was performed according to different tumor types and ICI drug types (Figure 4). Notably, in the PD-1 subgroup, male patients showed a better response to ICI treatment than female patients. However, in other drug types, this difference was not statistically significant. It is also noteworthy that the combination treatment modality of PD-1 with CTLA-4 was not significantly distinct between males and females. The difference in efficacy between sexes was also not significant in different tumor types. Additionally, we performed regression analyses on variables such as age and follow-up time, and the results showed that they were not associated with ICI efficacy in both males and females (Figure S4).

### 3.4. Effect of Sex on PFS and RFS after ICI Treatment

Besides OS, 33 and 12 studies used PFS and RFS as the survival outcome, respectively (Figures S5 and S6). We found ICI treatment improved survival outcomes of patients with cancer compared to controls, both in male patients (PFS: HR 0.63, 95% CI 0.58–0.69; RFS: HR 0.76, 95% CI 0.70–0.82) and female patients (PFS: HR 0.71, 95% CI 0.64–0.80; RFS: HR 0.74, 95% CI 0.67–0.82). The cumulative meta-analysis in Figures S7A,B and S8A,B and sensitivity analysis in Figures S7C,D and S8C,D showed relatively stable results. In contrast to OS, there was no significant difference between males and females in the efficacy of ICI treatment in terms of PFS (*p* = 0.10) and RFS (*p* = 0.79). In an interactional analysis of the sexes, ICI treatment improved the quality of survival of cancer patients more than controls (PFS: HR 0.66, 95% CI 0.62–0.71; RFS: HR 0.75, 95% CI 0.71–0.80), but there was higher heterogeneity between articles (PFS: I^2^ = 66%, *p* < 0.01; RFS: I^2^ = 45%, *p* < 0.01). Regression analysis for age and follow-up time showed absolutely no significant results (Figures S9 and S10).

## 4. Discussion

To the best of our knowledge, this study represents the most recent and comprehensive investigation into the impact of sex on ICI treatment outcomes. Fabio Conforti et al. [122] conducted the first meta-analysis of sex and ICI treatment effects in 2018, and found that males benefited more from ICI treatment than females. However, another study conducted by Fang Yang et al. [123] in 2020 came to a different conclusion, where there was no significant difference in the effect of ICI treatment between males and females. In a subsequent study, Li-Ting Lai et al. [19] also found no difference in results between the sexes. These conflicting findings impede a comprehensive understanding of the association between sex and ICI therapy. Nevertheless, it is worth noting that these studies only incorporated results from 20, 32 and 39 studies, respectively, consequently limiting the reliability of the conclusions drawn. Fortunately, with the abundance of recent clinical trials focusing on ICI drugs, we obtained more up-to-date trial data and incorporated them into our study for an updated meta-analysis. Hence, in this study, we analyzed 103 randomized controlled studies covering 66,582 study subjects.

The results of this study suggest that ICI therapy is an effective intervention with better efficacy than control treatment (conventional chemotherapy) for a wide range of tumor types. Among the results, males are more likely to benefit from ICI therapy than females, especially for anti-PD-1 types, as demonstrated by males exhibiting a lower risk of death (namely a better OS outcome). The results of this study are consistent with those of Fabio Conforti et al. [122].

The global increase in the burden of tumors poses a serious challenge to the health care system. Epidemiological analyses of tumors have identified sex differences as one of the factors influencing tumor incidence and mortality [124,125]; therefore, attempts have been made to explore the intrinsic associations between sex and tumors from different perspectives. Some researchers have analyzed these associations from a genetic aspect, starting from germline DNA or sex-dependent somatic mutations, in order to find possible oncogenic factors [126]. Also, differences in hormonal and immune systems between the sexes are thought to be closely related to tumor development. Many key immune-related genes are located on the X chromosome, thus innately determining that females have a stronger immune system to deal with the tumor challenge [1,127]. In addition, behavioral factors that differ between males and females in later life should also be included in assessments of the impact of tumors.

Immunotherapy that utilizes the body’s own immune system against cancer cells is an emerging field in oncology treatment. In particular, ICI therapies developed to address the immune escape mechanism of tumors are being used on a large scale in clinical practice. However, ICI therapy is not effective for all tumor patients, and requires consideration of the patient’s own condition. As a result of the influence of sex on the differences between the immune systems of males and females, it is readily thought that this may be an influential factor in the efficacy of ICI. As mentioned earlier, different studies on the relationship between sex and ICI efficacy have yielded conflicting results, leading to a lack of uniform understanding of whether it affects ICI efficacy.

Based on the results of our study on OS outcomes, males were more able to benefit from ICI treatment than females. This finding can be explained in terms of the following aspects. Firstly, since females tend to show stronger antitumor immunity than males [128], enhancing the immune system response via ICI therapy is less pronounced in females, which in turn manifests as a weaker response to ICI therapy than males. A recent study confirmed in mice that females show a stronger antitumor immune effect than males, and after treatment with ICI, male T-cell responses grew stronger, with a change that was greater than in females [129]. Secondly, there are differences between the sexes in the expressions of ICI-related receptors, such as PD-1 [130,131]. PD-1 is an immunosuppressive molecule. It has been shown that female tumor patients have higher serum and CD4+ T-cell membrane levels of PD-1 than males. Even though some studies have found sex differences in the efficacy of ICI in patients with equally high PD-1 expression [132], the effect of receptor expression differences between males and females can still not be excluded. It is worth noting that Xiangnan Guan et al. [133] recently identified androgen receptor (AR) as an essential element affecting the reactivity of prostate cancer patients to ICI therapy, and revealed the mechanism by which AR modulates antitumor immunity by regulating IFNγ activity. Combined PD-1 blockade and AR inhibition may result in better tumor suppression [133]. AR signaling has previously been identified as an important contributor to prostate cancer development, and AR blockade therapies have been widely used as an essential component of prostate cancer-targeted therapies [134]. The new study seems to provide a new insight into AR-targeted therapy from an immunological perspective, which is a win–win event for both prostate cancer treatment and tumor immunotherapy. Meanwhile, ChaoYang et al. [135] found that androgen receptor expression may be a potential factor contributing to sex differences in tumors. Through in vivo experiments and analysis of human samples, AR signaling was suggested to affect the activity of tumor-infiltrating CD8+ T cells, resulting in men exhibiting lower antitumor immune activity. When silencing the expression of the AR gene, the activity of CD8+ T cells was restored in male mice without similar changes in females. This result suggests that the level of AR is an important contributor to sex differences in antitumor immunity. Consistently, Hyunwoo Kwon et al. [136] also suggested that AR modulates the immune activity of CD8+ T cells in the tumor microenvironment, and leads to differences in tumor characteristics between males and females. Together, these studies uncover AR as a sex-differentiated tumor regulator that modulates antitumor immune activity. Also, the differences between sexes in tumor mutation burden [137], as well as exposure factors that differ later in life, lead to a stronger tumor mutation threat in males, which indirectly affects the therapeutic efficacy of ICI [138]. Meanwhile, a number of recent studies have focused on the effects of pregnancy on antitumor immunity. There have been reported promotive effects of pregnancy on regulatory immunity [139], and similar effects may persist long after pregnancy [140]. Fetal–maternal microchimerism and immune memory for shared cancer trophoblast antigens may be potential mechanisms of the effect [141]. In addition, we speculate that differences in the microbial environment between males and females may be an influential factor to be considered [142,143], although more adequate evidence is still needed to demonstrate the relationship between the two.

Different ICI drugs may influence the efficacy of treatment between the sexes. The effect of PD-1 treatment shows a more significant sex disparity than other treatment modalities. This finding differs from the findings of Yingcheng Wu et al. [144] and Antonino Grassadonia et al. [145], who found that CTLA-4 showed greater gender differences in efficacy than PD-1. This may be influenced by both tumor type and heterogeneity between articles, thus more targeted clinical studies on different ICI drug types may be necessary. In general, the various types of ICI drugs are almost universally more favorable to male patients in terms of their treatment efficacy. For tumor types, lung and gastrointestinal tumors have the highest number of RCT studies, which may be related to their high prevalence. An additional factor worth considering is that not all types of tumors respond well to ICI treatment. For example, ICI tends to be the best immunotherapy modality in lung cancer [146], whereas the benefits in uroepithelial tumors need to be further identified [147]. There does not seem to be a significant male–female difference in the therapeutic effect of ICI between different tumor types, a result that is consistent with the findings of Antonino Grassadonia et al. [143], but differs from that of Yingcheng Wu et al. [144]. In this case, the influence of differences in the tumor types themselves as well as quality variations of the studies should be considered. Variations in the number of studies may also contribute to biased results, thus more trials may be needed in the future to explore the benefits of ICI immunotherapy in different tumor types.

Consistently in terms of OS, ICI drugs had a favorable effect on the quality of survival in terms of the PFS and RFS of patients, but they did not seem to show a distinguishing effect between males and females. The effect of heterogeneity between articles needs to be taken into account for this, and due to the small number of studies, larger studies may be required to verify the reliability of the findings.

The major strength of this study is the inclusion of the most recent and comprehensive set of relevant clinical trials. In this research, we obtained 103 articles based on the results of RCT studies through an extensive literature search and a subsequent rigorous screening process, which provided a strong guarantee of the quality of the results. Also, the low publication bias among the included articles is another strength of this study. Meanwhile, during the subgroup analysis, we made a detailed classification of tumor types and treatment modalities separately, thus enabling more targeted study results and avoiding mutual interference between different tumor types or treatment modalities.

This study has various limitations. Firstly, our analysis was performed based on the results of previous studies rather than the raw data of the patients. The data for some important factors are inaccessible, such as receptor expression, posing difficulties for more comprehensive causal analyses. Meanwhile, we only performed subgroup analysis for different tumor types and treatment modalities. Despite these two being the most influential factors of interest to us, the possibility that the presence of other factors interfered with the difference in ICI treatment effect between sexes cannot be excluded. Moreover, we did not consider the effects of specific patterns of ICI use in our analysis, such as dosage and duration of treatment. For this reason, along with regional or ethnic differences in the populations enrolled in the studies, the heterogeneity that exists between their results is noteworthy. And, similar to other studies, the smaller numbers of female patients may have affected the accuracy of the results. The low number of female cases may be a result of the fact that there are fewer female tumors than male tumors originally [148], and most studies did not strictly require a female case size at the time of recruitment.

## 5. Conclusions

This systematic review and meta-analysis, comprising 103 randomized controlled trials, conclusively demonstrate a disparity in the sensitivity to ICI therapy between males and females. Specifically, our findings indicate that males tend to derive greater benefits from ICI treatment compared to females. This significant observation holds crucial implications for the field of oncology, considering the integral role of ICI therapy in cancer treatment and its positive impact on patient survival. However, in light of the differential response between male and female patients, it is imperative to consider sex differences when administering ICI therapy. Consequently, there may be a need to reconsider and tailor treatment regimens specifically for female patients. In this case, the different types of drugs or tumors is also one of the factors to be considered. Meanwhile, considering the lack of available clinical data on female ICI treatment, more female study subjects are needed to be included in subsequent studies to better guide the clinical application of ICI.

## Figures and Tables

**Table 1:** Characteristics of included studies in this systematic review and meta-analysis.

	Phase	Tumor Type	Line	Treatment Groups	Number of Patients	Number of Men (%)	Number of Women (%)	Median Age, Years	Median Follow-Up, Months	Outcome	Overall HR (95% CI)	HR (95% CI) for Men	HR (95% CI) for Women
Li-Tzong Chen et al. (2020) [10]	3	Gastrointestinal cancer	>1	nivolumab vs. placebo	493	348 (71%)	145 (29%)	61.7	27.3	OS	0.66 (0.54–0.80)	0.60 (0.48–0.76)	0.79 (0.55–1.15)
Alexander M M Eggermont et al. (2019) [11]	3	Melanoma	>1	ipilimumab vs. placebo	951	589 (62%)	362 (38%)	NA	82.8	OS	0.73 (0.60–0.90)	0.76 (0.55–1.05)	0.69 (0.45–1.07)
								NA	NA	RFS	0.76 (0.64–0.89)	0.76 (0.58–0.99)	0.76 (0.54–1.07)
M. Reck et al. (2021) [12]	3	NSCLC	1	nivolumab + ipilimumab + chemo vs. chemo	719	504 (70%)	215 (30%)	NA	30.7	OS	0.73 (0.61–0.87)	0.72 (0.59–0.88)	0.75 (0.54–1.04)
Masanobu Takahashi et al. (2021) [13]	3	Gastrointestinal cancer	>1	nivolumab vs. paclitaxel or docetaxel	274	230 (84%)	44 (16%)	NA	NA	OS	0.77 (0.59–1.01)	0.79 (0.59–1.06)	0.75 (0.37–1.55)
Hidetoshi Hayashi et al. (2022) [14]	2	NSCLC	>1	nivolumab vs. pemetrexed + carboplatin	102	43 (42%)	59 (58%)	NA	NA	PFS	1.92 (1.61–2.29)	1.92 (1.01–3.67)	1.79 (1.04–3.07)
Takashi Kojima et al. (2020) [15]	3	Gastrointestinal cancer	>1	pembrolizumab vs. chemo	628	544 (87%)	84 (13%)	NA	NA	PFS	0.89 (0.75–1.05)	0.89 (0.75–1.07)	0.90 (0.57–1.43)
Enriqueta Felip et al. (2021) [16]	3	NSCLC	>1	atezolizumab vs. best supportive care after adjuvant cisplatin-based chemo	1764	1178 (67%)	586 (33%)	NA	35.3	RFS	0.79 (0.64–0.96)	0.76 (0.59–0.99)	0.80 (0.57–1.13)
Makoto Nishio et al. (2021) [17]	3	NSCLC	1	atezolizumab + pemetrexed vs. pemetrexed	578	384 (66%)	194 (34%)	63.5 (31–85)	NA	PFS	0.60 (0.49–0.72)	0.64 (0.51–0.79)	0.51 (0.36–0.71)
										OS	0.86 (0.71–1.06)	0.93 (0.73–1.18)	0.76 (0.54–1.09)
Nancy Y Lee et al. (2021) [18]	3	HNC	>1	avelumab + chemo vs. placebo + chemo	697	575 (82%)	122 (18%)	NA	14.6 (8.5–19.6)	PFS	1.21 (0.93–1.57)	1.21 (0.91–1.61)	1.16 (0.58–2.33)
Keunchil Park et al. (2021) [19]	3	NSCLC	2	avelumab vs. docetaxel	529	367 (69%)	162 (31%)	NA	NA	OS	0.87 (0.71–1.05)	0.82 (0.65–1.03)	1.02 (0.72–1.45)
Caicun Zhou et al. (2021) [20]	3	NSCLC	>1	camrelizumab + carboplatin + pemetrexed vs. chemo	412	295 (72%)	117 (28%)	NA	11.9	PFS	0.60 (0.45–0.79)	0.55 (0.40–0.75)	0.78 (0.45–1.37)
D. R. Spigel et al. (2021) [21]	3	SCLC	>1	nivolumab vs. chemo	569	351 (62%)	218 (38%)	NA	15.8	OS	0.87 (0.73–1.05)	0.80 (0.63–1.01)	1.03 (0.77–1.38)
Caicun Zhou et al. (2022) [22]	3	NSCLC	1	sugemalimab vs. placebo	479	383 (80%)	96 (20%)	NA	8.6	PFS	0.48 (0.39–0.60)	0.48 (0.38–0.62)	0.6090.37–0.99)
F Stephen Hodi et al. (2010) [23]	3	Melanoma	>1	A: ipilimumab + gp100 vs. gp100	676	401 (59%)	275 (41%)	56.2 (NA)	21.0 vs. 27.8 vs. 17.2	OS	0.68 (NA)	0.66 (0.50–0.87)	0.72 (0.52–0.99)
				B: ipilimumab vs. gp100							0.66 (NA)	0.54 (0.37–0.77)	0.81 (0.55–1.20)
Antoni Ribas et al. (2013) [24]	3	Melanoma	1	tremelimumab vs. chemo	655	372 (57%)	283 (43%)	56.5 (22–90)	NA	OS	0.88 (NA)	0.93 (0.74–1.17)	0.81 (0.62–1.06)
Caroline Robert et al. (2011) [25]	3	Melanoma	1	lpilimumab + dacarbazine vs. dacarbazine + placebo	502	301 (60%)	201 (40%)	56.9 (NA)	54	OS	0.72 (0.59–0.87)	0.70 (0.55–0.90)	0.86 (0.63–1.17)
Ramaswamy Govindan et al. (2017) [26]	3	NSCLC	1	Ipilimumab + paclitaxel + carboplatin vs. paclitaxel + carboplatin + placebo	749	635 (85%)	114 (15%)	64 (28–85)	12.5 vs. 11.8	OS	0.91 (0.77–1.07)	0.85 (0.71–1.02)	1.33 (0.84–2.11)
Martin Reck et al. (2016) [27]	3	SCLC	1	Ipilimumab + etoposide + platinum vs. placebo + etoposide + platinum	954	643 (67%)	311 (33%)	62.5 (36–85)	10.5 vs. 10.2	OS	0.94 (0.81–1.09)	1.07 (0.89–1.28)	1.06 (0.81–1.37)
Julie Brahmer et al. (2015) [28]	3	NSCLC	>1	nivolumab vs. docetaxel	272	208 (76%)	64 (24%)	63 (39–85)	11	OS	0.59 (0.44–0.79)	0.57 (0.41–0.78)	0.67 (0.36–1.25)
Robert J Motzer et al. (2015) [29]	3	RCC	>1	nivolumab vs. everolimus	821	619 (75%)	202 (25%)	62 (18–88)	14	OS	0.73 (0.57–0.93)	0.73 (0.58–0.92)	0.84 (0.57–1.24)
Hossein Borghaei et al. (2015) [30]	3	NSCLC	>1	nivolumab vs. docetaxel	582	319 (55%)	263 (45%)	62 (21–85)	13.2	OS	0.73 (0.59–0.89)	0.73 (0.56–0.96)	0.78 (0.58–1.04)
James Larkin et al. (2018) [31]	3	Melanoma	>1	nivolumab vs. chemo	405	261 (64%)	144 (36%)	60 (23–88)	NA	OS	0.92 (0.71–1.18)	0.85 (0.62–1.17)	1.07 (0.69–1.65)
Caroline Robert et al. (2015) [32]	3	Melanoma	1	nivolumab vs. dacarbazine	418	246 (59%)	172 (41%)	65 (18–87)	8.9 vs. 6.8	OS	0.42 (0.25–0.73)	0.34 (0.22–0.54)	0.56 (0.33–0.95)
Michele Maio et al. (2017) [33]	2b	Others	>1	tremelimumab vs. placebo	571	434 (76%)	137 (24%)	66.5 (60–73)	NA	OS	0.92 (0.76–1.12)	0.91 (0.73–1.13)	1.12 (0.72–1.75)
Roy S Herbst et al. (2016) [34]	2/3	NSCLC	>1	pembrolizumab vs. docetaxel	1033	634 (61%)	399 (39%)	62.5 (54–70)	13.1	PFS	0.85 (0.73–0.98)	0.78 (0.64–0.94)	1.02 (0.78–1.32)
Roy S. Herbst et al. (2021) [35]	2/3	NSCLC	>1	pembrolizumab vs. docetaxel	1033	634 (61%)	399 (39%)	NA	67.4	OS	0.70 (0.61–0.80)	0.71 (0.60–0.86)	0.66 (0.53–0.84)
Louis Fehrenbacher et al. (2018) [36]	3	NSCLC	>1	atezolizumab vs. docetaxel	1225	467 (38%)	758 (62%)	63.5 (25–85)	28	OS	0.80 (0.70–0.92)	0.81 (0.65–1.01)	0.79 (0.66–0.93)
David P Carbone et al. (2017) [37]	3	NSCLC	1	nivolumab vs. chemo	541	332 (61%)	209 (39%)	64 (29–89)	13.5	OS	1.02 (0.80–1.30)	0.97 (0.74–1.26)	1.15 (0.79–1.66)
Robert L Ferris et al. (2016) [38]	3	HNC	>1	nivolumab vs. chemo	361	300 (83%)	61 (17%)	60 (28–83)	5.1	OS	0.70 (0.51–0.96)	0.65 (0.48–0.88)	0.93 (0.47–1.85)
Scott J Antonia et al. (2018) [39]	3	NSCLC	>1	durvalumab vs. placebo	713	500 (70%)	213 (30%)	NA	25.2	OS	0.68 (0.54–0.86)	0.78 (0.59–1.03)	0.46 (0.30–0.73)
Pier Francesco Ferrucci et al. (2020) [40]	2	Melanoma	>1	pembrolizumab + dabrafenib + trametinib vs. placebo + dabrafenib + trametinib	120	69 (58%)	51 (43%)	56 (18–83)	36.6	PFS	0.53 (0.34–0.83)	0.45 (0.25–0.82)	0.79 (0.41–1.53)
										OS	0.64 (0.38–1.06)	0.62 (0.31–1.25)	0.64 (0.30–1.38)
Martin Reck et al. (2019) [41]	3	NSCLC	1	pembrolizumab vs. platinum-based chemo	305	187 (61%)	118 (39%)	65.2 (33–90)	25.2	OS	0.63 (0.47–0.86)	0.54 (0.36–0.79)	0.95 (0.56–1.62)
Tony S K Mok et al. (2019) [42]	3	NSCLC	1	pembrolizumab vs. chemo	1274	902 (71%)	372 (29%)	63 (57–69)	12.8	OS	0.81 (0.71–0.93)	0.80 (0.68–0.94)	0.89 (0.68–1.17)
Robert J Motzer et al. (2022) [43]	3	RCC	1	nivolumab + ipilimumab vs. sunitinib	636	477 (75%)	159 (25%)	62 (21–85)	67.7	OS	0.94 (0.65–1.37)	0.81 (0.68–0.97)	0.62 (0.45–0.84)
Ezra E W Cohen et al. (2019) [44]	3	HNC	>1	pembrolizumab vs. standard of care	495	412 (83%)	83 (17%)	60.0 (54–66)	7.5	OS	0.80 (0.65–0.98)	0.77 (0.62–0.96)	0.94 (0.54–1.63)
Joaquim Bellmunt et al. (2017) [45]	3	Urothelial carcinoma	>1	pembrolizumab vs. chemo	542	402 (74%)	140 (26%)	66 (26–88)	14.1	OS	0.73 (0.59–0.91)	0.73 (0.56–0.94)	0.78 (0.49–1.24)
Barbara Burtness et al. (2019) [46]	3	HNC	1	A: pembrolizumab vs. cetuximab + chemo	601	511 (85%)	90 (15%)	61.3 (54.5–68)	11.5 vs. 13.0 vs. 10.7	OS	0.81 (0.68–0.97)	0.80 (0.66–0.97)	0.89 (0.56–1.41)
				B: pembrolizumab + chemo vs. cetuximab + chemo	559	466 (83%)	93 (17%)				0.72 (0.60–0.86)	0.72 (0.59–0.89)	0.67 (0.42–1.06)
Alexander M M Eggermont et al. (2021) [47]	3	Melanoma	>1	pembrolizumab vs. placebo	1019	628 (62%)	391 (38%)	NA	42.3	PFS	0.60 (0.49–0.73)	0.56 (0.41–0.77)	0.68 (0.44–1.05)
Martin Reck et al. (2019) [48]	3	NSCLC	1	atezolizumab + bevacizumab + carboplatin + paclitaxel vs. bevacizumab + carboplatin + paclitaxel	800	479 (60%)	321 (40%)	63 (31–90)	19.6 vs. 19.7	OS	0.76 (0.63–0.93)	0.73 (0.57–0.93)	0.82 (0.61–1.12)
Howard West et al. (2019) [49]	3	NSCLC	1	atezolizumab + carboplatin + nabpaclitaxel vs. chemo	679	400 (59%)	279 (41%)	64.3 (18–86)	18.5 vs. 19.2	OS	0.80 (0.65–0.99)	0.87 (0.66–1.15)	0.66 (0.46–0.93)
Robert Jotte et al. (2020) [50]	3	NSCLC	1	atezolizumab + carboplatin + nab-paclitaxel vs. carboplatin + nab-paclitaxel	683	557 (82%)	126 (18%)	65 (23–83) vs. 65 (38–86)	26.8 vs. 24.8	OS	0.88 (0.73–1.05)	0.91 (0.75–1.12)	0.68 (0.44–1.04)
										PFS	0.71 (0.60–0.85)	0.71 (0.59–0.85)	0.66 (0.45–0.97)
Charles S. Fuchs et al. (2022) [51]	3	Gastrointestinal cancer	>1	pembrolizumab vs. paclitaxel	395	286 (72%)	109 (28%)	NA	NA	OS	0.81 (0.66–1.00)	0.87 (0.68–1.11)	0.77 (0.50–1.18)
Fabrice Barlesi et al. (2018) [52]	3	NSCLC	>1	avelumab vs. docetaxel	529	367 (69%)	162 (31%)	63.5 (56–70)	18.3	OS	0.90 (0.73–1.12)	0.83 (0.64–1.08)	1.08 (0.74–1.59)
Roy S Herbst et al. (2020) [53]	3	NSCLC	1	atezolizumab vs. chemo	205	143 (70%)	62 (30%)	NA	NA	OS	0.59 (0.40–0.89)	0.57 (0.35–0.93)	0.69 (0.34–1.39)
Joaquim Bellmunt et al. (2021) [54]	3	Urothelial carcinoma	>1	atezolizumab vs. observation	809	638 (79%)	171 (21%)	NA	21.9	RFS	0.89 (0.74–1.08)	0.91 (0.73–1.13)	1.00 (0.79–1.29)
Naiyer A Rizvi et al. (2020) [55]	3	NSCLC	1	durvalumab vs. chemo	325	219 (67%)	106 (33%)	64.2 (32–85)	30.2	OS	0.76 (0.56–1.02)	0.81 (0.59–1.11)	0.66 (0.41–1.04)
Luis G Paz-Ares et al. (2022) [56]	3	NSCLC	1	nivolumab + ipilimumab vs. chemo	1166	778 (67%)	388 (33%)	NA	54.8	OS	0.74 (0.65–0.84)	0.67 (0.57–0.78)	0.88 (0.70–1.11)
Hirotsugu Kenmotsu et al. (2022) [57]	3	NSCLC	>1	atezolizumab vs. best supportive care	74	58 (78%)	16 (22%)	64.0 (40–75) vs. 68.0 (37–74)	38.3	RFS	0.52 (0.25–1.08)	0.50 (0.21–1.17)	0.66 (0.16–2.73)
Kohei Shitara et al. (2020) [58]	3	Gastrointestinal cancer	1	A: pembrolizumab vs. chemo	506	359 (71%)	147 (29%)	61.7 (20–87)	29.4	OS	0.91 (0.74–1.1)	0.88 (0.70–1.11)	0.90 (0.63–1.27)
				B: pembrolizumab + chemo vs. chemo	507	374 (74%)	133 (26%)	62.2 (22–87)		OS	0.85 (0.7–1.03)	0.84 (0.67–1.05)	0.89 (0.62–1.29)
Mary O’Brien et al. (2022) [59]	3	NSCLC	>1	Pembrolizumab vs. placebo	1177	804 (68%)	373 (32%)	65 (59–70)	35.6	RFS	0.76 (0.63–0.91)	0.81 (0.65–1.01)	0.73 (0.54–1.00)
Kenneth F Grossmann et al. (2022) [60]	3	Melanoma	>1	pembrolizumab vs. standard of care	1201	678 (56%)	523 (44%)	53 (20, 82) vs. 54 (18, 86)	47.5	OS	0.82 (0.62–1.07)	0.7 (0.43–1.15)	0.87 (0.63–1.21)
										RFS	0.76 (0.64–0.91)	0.75 (0.55–1.03)	0.76 (0.62–0.93)
Thomas Powles et al. (2020) [61]	3	Urothelial carcinoma	1	durvalumab + tremelimumab vs. chemo	686	530 (77%)	156 (23%)	68 (60–73)	41.2	OS	0.85 (0.72–1.02)	0.84 (0.69–1.02)	0.90 (0.63–1.30)
Elisabeth Livingstone et al. (2022) [62]	2	Melanoma	>1	A: nivolumab + ipilimumab vs. placebo	108	64 (59%)	44 (41%)	55.0 (46.0–65.0)	49.2	RFS	0.25 (0.13–0.48)	0.33 (0.17–0.66)	0.17 (0.07–0.44)
				B: nivolumab vs. placebo	111	64 (58%)	47 (42%)	55.0 (46.0–65.1)	49.2	RFS	0.60 (0.36–1.00)	0.80 (0.45–1.41)	0.43 (0.21–0.87)
Taofeek K Owonikoko et al. (2021) [63]	3	SCLC	>1	A: chemo + nivolumab + ipilimumab	554	355 (64%)	199 (36%)	NA	NA	OS	0.92 (0.76–1.12)	0.99 (0.78–1.27)	0.82 (0.58–1.14)
				B: chemo +nivolumab vs. chemo + placebo	555	352 (63%)	203 (37%)			OS	0.83 (0.63–1.01)	0.89 (0.70–1.14)	0.75 (0.54–1.04)
Luis A Diaz Jr. et al. (2022) [64]	3	Gastrointestinal cancer	1	pembrolizumab vs. mFOLFOX6	307	153 (50%)	154 (50%)	63 (50–73)	NA	OS	0.74 (0.53–1.03)	0.61 (0.38–0.99)	0.88 (0.55–1.41)
Thierry André et al. (2020) [65]	3	Gastrointestinal cancer	1	pembrolizumab vs. chemo	307	153 (50%)	154 (50%)	63 (24–93)	32.4	PFS	0.60 (0.45–0.80)	0.59 (0.38–0.90)	0.58 (0.39–0.87)
Ken Kato et al. (2019) [66]	3	Gastrointestinal cancer	2	nivolumab vs. chemo	419	364 (87%)	55 (13%)	65.5 (57–72)	10.5 vs. 8.0	OS	0.77 (0.62–0.95)	0.79 (0.63–0.99)	0.72 (0.38–1.36)
D Rodríguez-Abreu et al. (2021) [67]	3	NSCLC	1	pemetrexed + platinum + pembrolizumab vs. pemetrexed + platinum	616	363 (59%)	253 (41%)	64.5 (34–84)	31	OS	0.56 (0.46–0.69)	0.74 (0.56–0.96)	0.41 (0.30–0.56)
										PFS	0.49 (0.41–0.59)	0.58 (0.46–0.74)	0.39 (0.29–0.52)
Thomas Powles et al. (2020) [68]	3	Urothelial carcinoma	>1	avelumab + best support care vs. best support care	700	541 (77%)	159 (23%)	68.5 (32–90)	NA	OS	0.69 (0.56–0.86)	0.64 (0.50–0.83)	0.89 (0.56–1.41)
										PFS	0.62 (0.52–0.75)	0.60 (0.49–0.74)	0.69 (0.47–1.01)
Markus Moehler et al. (2021) [69]	3	Gastrointestinal cancer	1	avelumab vs. chemo	499	331 (66%)	168 (34%)	NA	NA	OS	0.90 (0.74–1.11)	0.83 (0.64–1.07)	1.06 (0.76–1.49)
Y-J Bang et al. (2018) [70]	3	Gastrointestinal cancer	>1	avelumab vs. chemo	371	267 (72%)	104 (28%)	60 (18–86)	10.6	OS	1.1 (0.90–1.40)	0.99 (0.75–1.32)	1.54 (0.99–2.38)
D.F. Bajorin et al. (2021) [71]	3	Urothelial carcinoma	>1	nivolumab vs. placebo	709	540 (76%)	169 (24%)	NA	NA	RFS	0.70 (0.57–0.86)	0.68 (0.54–0.87)	0.76 (0.50–1.16)
T. K. Choueiri et al. (2020) [72]	3	RCC	1	avelumab + axitinib vs. sunitinib	886	660 (74%)	226 (26%)	NA	NA	PFS	0.688 (0.574–0.825)	0.647 (0.524–0.798)	0.862 (0.604–1.229)
										OS	0.796 (0.616- 1.027)	0.797 (0.594–1.071)	0.814 (0.486–1.364)
Xiaofei Zhu et al. (2022) [73]	2	Gastrointestinal cancer	>1	stereotactic body radiotherapy + pembrolizumab + trametinib vs. SBRT + gemcitabine	170	105 (62%)	65 (38%)	NA	13.1	OS; PFS	OS: 0.69 (0.51–0.95); PFS: 0.60 (0.44–0.81)	OS: 0.62 (0.42–0.92); PFS: 0.52 (0.35–0.77)	OS: 0.86 (0.52–1.40); PFS: 0.76 (0.47–1.24)
R.J. Kelly et al. (2021) [74]	3	Gastrointestinal cancer	>1	nivolumab vs. placebo	532	671 (126%)	123 (23%)	NA	24.4	RFS	0.70 (0.58–0.86)	0.73 (0.59–0.91)	0.59 (0.35–1.00)
Yoon-Koo Kang et al. (2022) [75]	2/3	Gastrointestinal cancer	>1	nivolumab + chemo vs. placebo + chemo	724	523 (72%)	201 (28%)	NA	11.6	PFS	0.68 (0.51–0.90)	0.70 (0.54–0.90)	0.72 (0.48–1.10)
										OS	0.90 (0.75–1.08)	0.87 (0.70–1.07)	0.99 (0.70–1.39)
Stephen V. Liu et al. (2021) [76]	1/3	SCLC	1	carboplatin + etoposide (CP/ET) + atezolizumab vs. CP/ET + placebo	403	261 (65%)	142 (35%)	NA	NA	OS	0.76 (0.60–0.95)	0.83 (0.63–1.10)	0.64 (0.43–0.94)
Luis Paz-Ares et al. (2018) [77]	3	NSCLC	1	pembrolizumab + chemo vs. placebo + chemo	559	455 (81%)	104 (19%)	65 (29–88)	7.8	OS	0.64 (0.49–0.85)	0.69 (0.51–0.94)	0.42 (0.22–0.81)
Matthew D Galsky et al. (2020) [78]	3	Urothelial carcinoma	>1	atezolizumab + chemo vs. chemo	1213	636 (52%)	215 (18%)	69 (62–75) vs. 67 (61–73)	11.8	OS	0.84 (0.69–1.00)	0.83 (0.67–1.02)	0.88 (0.61–1.26)
										PFS	0.81 (0.69–0.94)	0.83 (0.69–0.99)	0.75 (0.55–1.02)
R. Motzer et al. (2021) [79]	3	RCC	>1	lenvatinib + pembrolizumab vs. sunitinib	712	530 (74%)	209 (26%)	NA	NA	PFS	0.39 (0.32–0.49)	0.38 (0.30–0.49)	0.42 (0.27–0.66)
Eric X. Chen et al. (2020) [80]	2	Gastrointestinal cancer	>1	tremelimumab + durvalumab + BSC vs. BSC	180	121 (67%)	59 (33%)	65 (36–87)	15.2	OS	0.72 (0.54–0.97)	0.79 (0.57–1.10)	0.55 (0.32–0.95)
Tianshu Liu et al. (2022) [81]	3	Gastrointestinal cancer	1	nivolumab + chemo vs. chemo	156	105 (67%)	51 (33%)	60.5 (23–85)	14.0 vs. 9.9	OS	0.54 (0.37–0.79)	0.52 (0.33–0.83)	0.68 (0.30–1.11)
Daniel J. Renouf et al. (2022) [82]	2	Gastrointestinal cancer	>1	gemcitabine + nab-paclitaxel + durvalumab + tremelimumab vs. gemcitabine + nab-paclitaxe	180	93 (52%)	87 (48%)	64.3 (29–84)	28.5	OS	0.94 (0.71–1.25)	1.00 (0.66–1.64)	0.84 (0.57–1.26)
S. Peters et al. (2022) [83]	3	Others	1	nivolumab + ipilimumab vs. chemo	605	467 (77%)	138 (23%)	69 (62–75)	43.1	OS	0.75 (0.63–0.90)	0.73 (0.60–0.90)	0.82 (0.56–1.20)
Ralf Gutzmer et al. (2020) [84]	3	Melanoma	1	atezolizumab + vemurafenib + cobimetinib vs. placebo + vemurafenib + cobimetinib	514	299 (58%)	215 (42%)	54.0 (44.8–64.0) vs. 53.5 (43.0–63.8)	18.9	PFS	0.77 (0.62–0.96)	0.75 (0.56–1.01)	0.81 (0.58–1.13)
S. Popat et al. (2020) [85]	3	Others	>1	pembrolizumab vs. chemo	144	118 (82%)	26 (18%)	NA	NA	OS	1.11 (0.73–1.66)	1.16 (0.74–1.82)	0.91 (0.34–2.45)
Sumanta Kumar Pal et al. (2022) [86]	3	RCC	>1	atezolizumab vs. placebo	778	565 (73%)	213 (27%)	60 (52–69)	45	RFS	0.93 (0.75–1.15)	1.08 (0.84–1.39)	0.61 (0.40–0.94)
L. Paz-Ares et al. (2022) [87]	3	SCLC	1	A: durvalumab + EP vs. EP	537	374 (70%)	163 (30%)	NA	39.4	OS	0.71 (0.60–0.86)	0.76 (0.62–0.95)	0.60 (0.42–0.84)
				B: durvalumab + tremelimumab + EP vs. EP	537	386 (72%)	151 (28%)	NA	39.4	OS	0.81 (0.67–0.97)	0.81 (0.65–1.00)	0.74 (0.52–1.05)
Jean-Louis Pujol et al. (2019) [88]	2	SCLC	2	atezolizumab vs. chemo	73	43 (59%)	30 (41%)	64.7 (51.1–85.5)	13.7	OS	0.84 (0.45–1.58)	0.82 (0.36–1.91)	0.74 (0.26–2.07)
Dean A. Fennell et al. (2021) [89]	3	Others	>1	nivolumab vs. placebo	332	253 (76%)	79 (24%)	NA	11.6	PFS	0.67 (0.53–0.85)	0.65 (0.49–0.86)	0.69 (0.42–1.14)
										OS	0.69 (0.52–0.91)	0.63 (0.46–0.87)	0.96 (0.51–1.79)
Charles M Rudin et al. (2020) [90]	3	SCLC	1	pembrolizumab + EP vs. placebo + EP	453	294 (65%)	159 (35%)	64.5 (24–83)	NA	PFS	0.73 (0.60–0.88)	0.66 (0.52–0.84)	0.76 (0.54–1.06)
										OS	0.80 (0.64–0.98)	0.76 (0.59–0.98)	0.88 (0.61–1.26)
Ahmet Sezer et al. (2021) [91]	3	NSCLC	1	cemiplimab vs. platinum-doublet chemo	463	479 (103%)	84 (18%)	NA	NA	OS	0.57 (0.42–0.77)	0.50 (0.36–0.69)	1.11 (0.49–2.52)
										PFS	0.54 (0.43–0.68)	0.50 (0.40–0.64)	0.79 (0.43–1.46)
Jing Huang et al. (2020) [92]	3	Gastrointestinal cancer	2	camrelizumab vs. chemo	448	400 (89%)	48 (11%)	60 (54–65)	8.3 vs. 6.2	OS	0.71 (0.57–0.87)	0.75 (0.60–0.93)	0.45 (0.21–0.93)
S. Sugawara et al. (2021) [93]	3	NSCLC	1	nivolumab + carboplatin, paclitaxel and bevacizumab vs. chemo + carboplatin, paclitaxel and bevacizumab	550	411 (75%)	139 (25%)	NA	NA	PFS	0.57 (0.46–0.72)	0.53 (0.41–0.69)	0.72 (0.45–1.15)
Thomas Powles et al. (2022) [94]	3	RCC	>1	pembrolizumab vs. placebo	994	706 (71%)	288 (29%)	NA	30.1	RFS	0.63 (0.50–0.80)	0.60 (0.45–0.80)	0.73 (0.48–1.13)
Michael Boyer et al. (2021) [95]	3	NSCLC	>1	ipilimumab vs. saline placebo	568	393 (69%)	174 (31%)	NA	NA	OS	1.08 (0.85–1.37)	0.97 (0.73–1.29)	1.29 (0.84–1.99)
										PFS	1.06 (0.86–1.30)	1.02 (0.80–1.29)	1.15 (0.78–1.69)
Caicun Zhou et al. (2022) [96]	3	NSCLC	>1	tislelizumab vs. docetaxel	805	622 (77%)	183 (23%)	NA	16	OS	0.66 (0.56–0.79)	0.61 (0.50–0.74)	0.95 (0.65–1.38)
Richard S. Finn et al. (2020) [97]	3	Gastrointestinalcancer	>1	atezolizumab + bevacizumab vs. sorafenib	501	414 (83%)	87 (17%)	NA	NA	OS	0.60 (0.44–0.82)	0.66 (0.47–0.92)	0.35 (0.15–0.81)
										PFS	0.59 (0.47–0.75)	0.59 (0.45–0.77)	0.60 (0.34–1.06)
Hai-Qiang Mai et al. (2021) [98]	3	Others	1	toripalimab + GP vs. placebo + GP	289	240 (83%)	49 (17%)	48 (19–72)	NA	PFS	0.51 (0.356–0.728)	0.54 (0.360–0.806)	0.41 (0.187–0.889)
Jie Wang et al. (2021) [99]	3	NSCLC	>1	A: tislelizumab + PC vs. PC	241	218 (90%)	23 (10%)	62 (34–74)	NA	PFS	0.52 (0.37–0.74)	0.53 (0.37–0.76)	0.53 (0.17–1.61)
				B: tislelizumab + nab-PC vs. PC	240	223 (93%)	17 (7%)				0.48 (0.34–0.68)	0.50 (0.35–0.71)	0.36 (0.09–1.47)
Li Zhang et al. (2022) [100]	3	NSCLC	1	sintilimab + chemo vs. placebo + chemo	397	303 (76%)	94 (24%)	NA	30.8	OS	0.65 (0.50–0.85)	0.57 (0.43–0.77)	0.99 (0.56–1.77)
Yunpeng Yang et al. (2020) [101]	3	NSCLC	1	sintilimab + chemo vs. placebo + chemo	397	303 (76%)	94 (24%)	61 (30, 75)	8.9	PFS	0.482 (0.362–0.643)	0.443 (0.320–0.612)	0.603 (0.332–1.098)
Huiyan Luo et al. (2021) [102]	3	Gastrointestinal cancer	>1	camrelizumab + chemo vs. placebo + chemo	596	523 (88%)	73 (12%)	NA	10.8	OS	0.70 (0.56–0.88)	0.69 (0.55–0.88)	0.87 (0.42–1.81)
Jie Wang et al. (2022) [103]	3	SCLC	1	adebrelimab + chemo vs. placebo + chemo	462	372 (81%)	90 (19%)	62 (56–66)	13.5	OS	0.72 (0.58–0.90)	0.72 (0.57–0.92)	0.62 (0.37–1.05)
Qing Zhou et al. (2022) [104]	3	NSCLC	>1	sugemalimab vs. placebo	381	351 (92%)	30 (8%)	NA	NA	PFS	0.64 (0.48–0.85)	0.61 (0.45–0.82)	1.40 (0.55–3.57)
Shun Lu et al. (2022) [105]	3	NSCLC	>1	sintilimab + bevacizumab biosimilar IBI305 + pemetrexed + cisplatin vs. pemetrexed + cisplatin	299	123 (41%)	176 (59%)	NA	NA	PFS	0.46 (0.34–0.64)	0.67 (0.41–1.09)	0.44 (0.30–0.66)
Zi-Xian Wang et al. (2022) [106]	3	Gastrointestinal cancer	>1	toripalimab + TP vs. placebo + TP	514	437 (85%)	77 (15%)	NA	NA	PFS	0.57 (0.45–0.72)	0.51 (0.40–0.66)	0.96 (0.53–1.75)
										OS	0.59 (0.43–0.80)	0.50 (0.36–0.70)	1.40 (0.60–3.28)
Ying Cheng et al. (2022) [107]	3	SCLC	1	serplulimab + chemo vs. placebo + chemo	585	481 (82%)	104 (18%)	61.1	12.3	OS	0.63 (0.49–0.82)	0.64 (0.48–0.84)	0.57 (0.30–1.06)
Miranda Gogishvili et al. (2022) [108]	3	NSCLC	1	cemiplimab + chemo vs. placebo + chemo	312	268 (86%)	44 (14%)	63.0 (57–68)	16.3 vs. 16.7	OS	0.71 (0.53–0.93)	0.55 (0.41–0.74)	2.11 (0.89–5.03)
										PFS	0.56 (0.44–0.70)	0.48 (0.37–0.61)	0.90 (0.50–1.62)
Alexander M. M. Eggermont et al. (2020) [109]	3	Melanoma	>1	pembrolizumab vs. placebo	1019	628 (62%)	391 (38%)	54 (19–88)	36.6	RFS	0.56 (0.47–0.68)	0.55 (0.40–0.74)	0.60 (0.40–0.90)
Richard S. Finn et al. (2020) [110]	3	Gastrointestinal cancer	>1	pembrolizumab + BSC vs. placebo + BSC	413	338 (82%)	75 (18%)	13.8	NA	OS	0.78 (0.61–1.00)	0.76 (0.58–1.00)	0.80 (0.44–1.47)
										PFS	0.72 (0.57–0.90)	0.74 (0.57–0.95)	0.59 (0.33–1.06)
F. Stephen Hodi et al. (2016) [111]	2	Melanoma	>1	nivolumab and ipilimumab vs. ipilimumab	142	95 (67%)	47 (33%)	65	24.5	OS	0.74 (0.43–1.26)	0.65 (0.33–1.26)	0.89 (0.36–2.19)
Brian I. Rini et al. (2019) [112]	3	RCC	>1	pembrolizumab + axitinib vs. sunitinib	861	628 (73%)	233 (27%)	61.5	12.8	OS	0.53 (0.38–0.74)	0.54 (0.37–0.80)	0.45 (0.25–0.83)
										PFS	0.69 (0.57–0.84)	0.77 (0.61–0.97)	0.54 (0.37–0.81)

BSC: best supportive care; EP: carboplatin or cisplatin + etoposide; TP: paclitaxel + cisplatin.

## Supplementary Materials

The following supporting information can be downloaded at: https://www.mdpi.com/article/10.3390/cancers16020382/s1. Figure S1: Cumulative meta-analysis and Sensitivity analysis for HR of males, with OS as the outcome; Figure S2: Cumulative meta-analysis and Sensitivity analysis for HR of females, with OS as the outcome; Figure S3: Subgroup analysis of hazard ratios for death in tumor patients stratified by tumor type and ICI drug type, by sex, with OS as the outcome; Figure S4: Meta-regression for HR of males and females, with OS as the outcome; Figure S5: Hazard ratios for death in the intervention and control groups in males (A) and females (B), with PFS as the outcome; Figure S6: Hazard ratios for death in the intervention and control groups in males (A) and females (B), with RFS as the outcome; Figure S7: Cumulative meta-analysis and Sensitivity analysis for HR with PFS as the outcome; Figure S8: Cumulative meta-analysis and Sensitivity analysis for HR with RFS as the outcome; Figure S9: Meta-regression for HR of males and females, with PFS as the outcome; Figure S10: Meta-regression for HR of males and females, with RFS as the outcome; Table S1: PRISMA 2020 Checklist; Table S2: Details of Search Strategy to Retrieve the Studies using PubMed (Medline). Date of Search: 11/28/2023; Table S3: Details of Search Strategy to Retrieve the Studies using Embase. Date of Search: 11/28/2023; Table S4: Details of Search Strategy to Retrieve the Studies using Cochrane. Date of Search: 11/28/2023; Table S5: Details of Search Strategy to Retrieve the Studies using Web of Science. Date of Search: 11/28/2023; Table S6: Jadad quality score of included studies.
