# Supplementary material for: The Effect of Sex on the Therapeutic Efficiency of Immune Checkpoint Inhibitors: A Systematic Review and Meta-Analysis Based on Randomized Controlled Trials
Source: Cancers (Basel). 2024 Jan 16;16(2):382. doi: 10.3390/cancers16020382 (PMC10814446; doi:10.3390/cancers16020382)
Supplement: Supplementary file 1 [file cancers-16-00382-s001.zip › cancers-2789091-supplementary.pdf]

Supplementary Material

The effect of sex on the therapeutic efficiency of immune checkpoint inhibitors: A systematic review and meta-analysis based on randomized controlled trials

Xing-Yu Zhong, Jian-Xuan Sun, Na Zeng, Yi-Fan Xiong, Ye An, Shao-Gang Wang*, Qi-Dong Xia*

*** Correspondence:**

Shao-Gang Wang: sgwangtjm@163.com

Qi-Dong Xia: qidongxia_md@163.com;

1 Supplementary Figures and Tables

1.1 Supplementary Figures

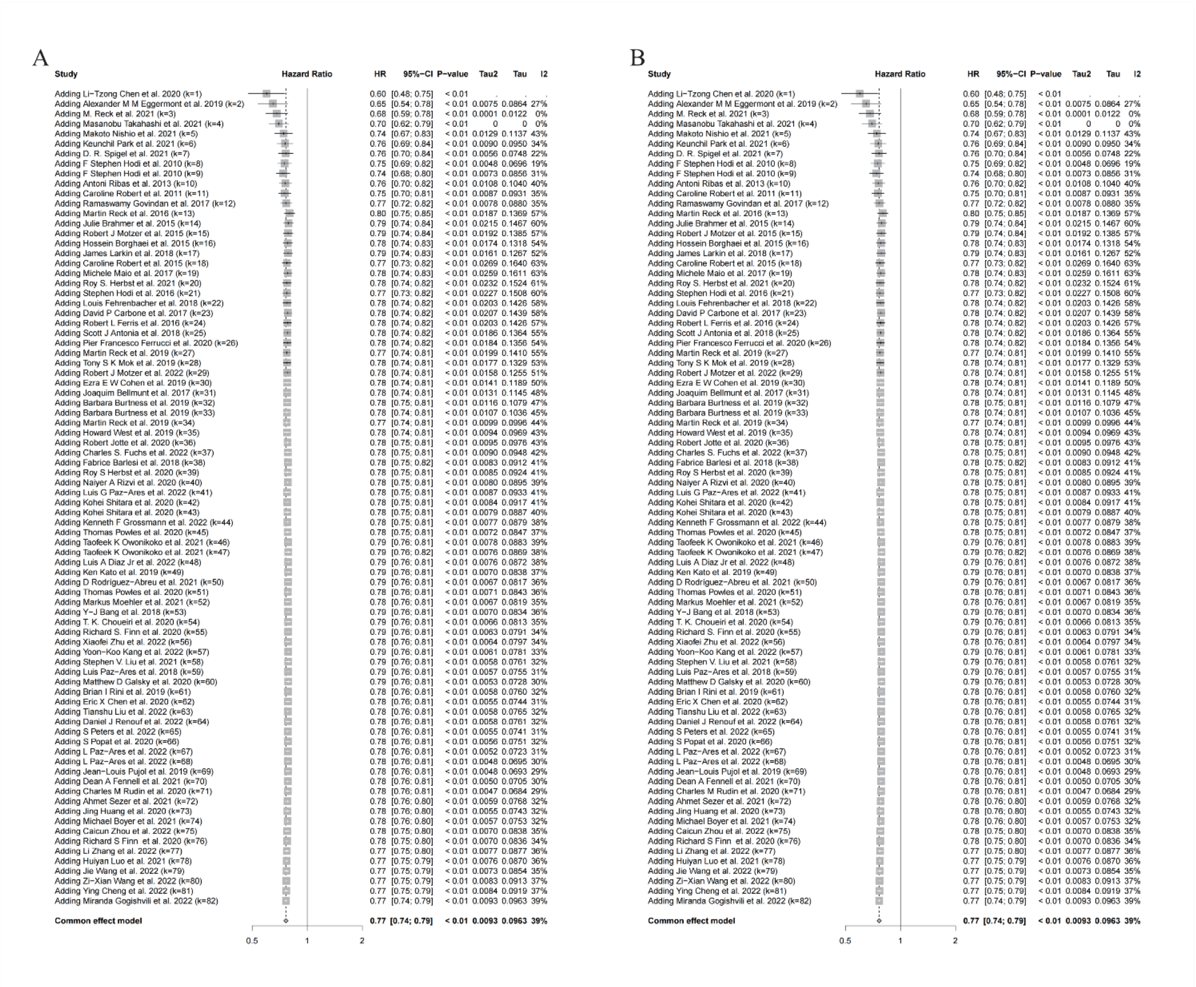

Figure S1. Cumulative meta-analysis and Sensitivity analysis for HR of males, with OS as the outcome. A) Cumulative meta-analysis by stepwise adding the included studies. B) Sensitivity analysis by stepwise omitting the included studies.

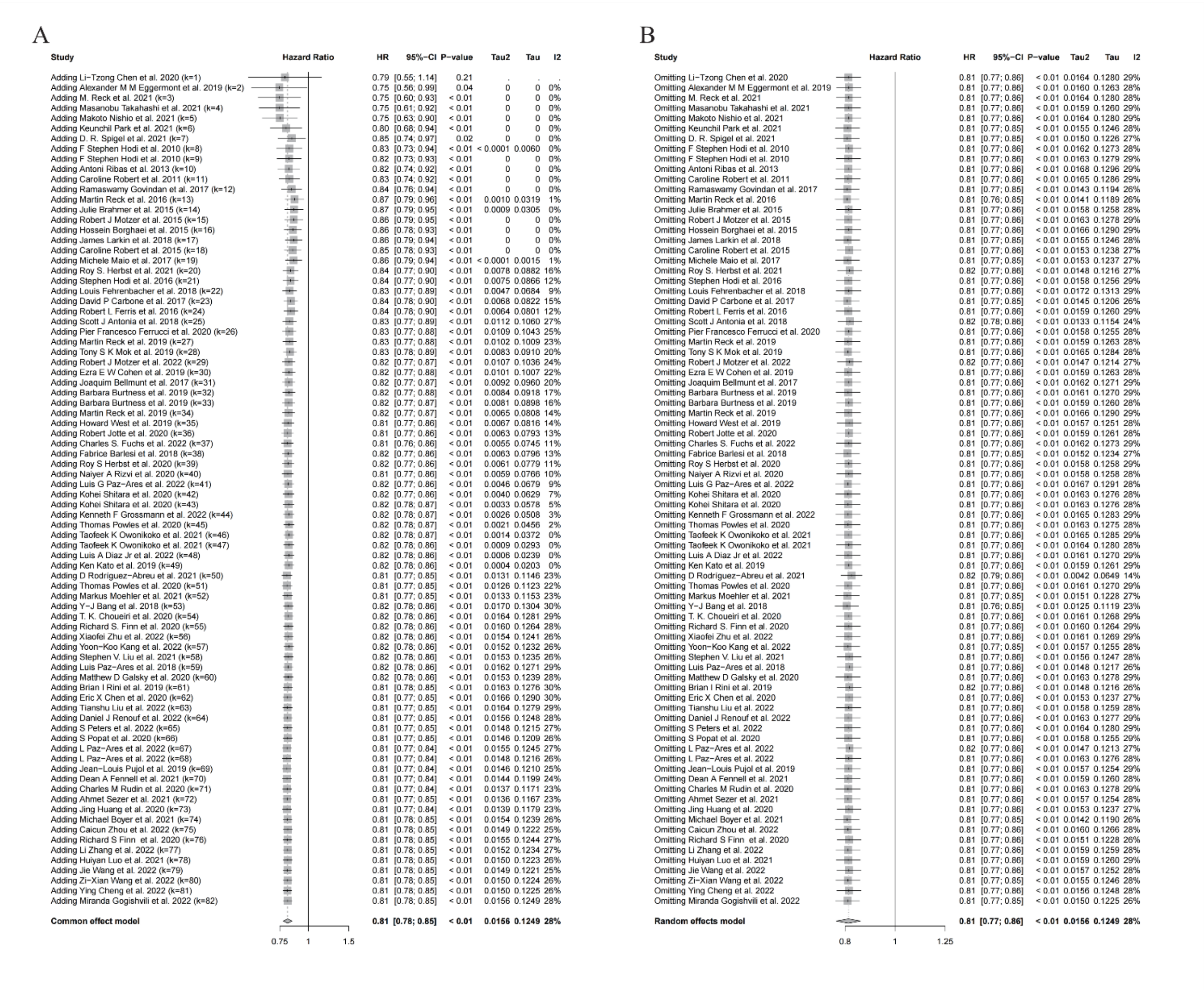

Figure S2. Cumulative meta-analysis and Sensitivity analysis for HR of females, with OS as the outcome. A) Cumulative meta-analysis by stepwise adding the included studies. B) Sensitivity analysis by stepwise omitting the included studies.

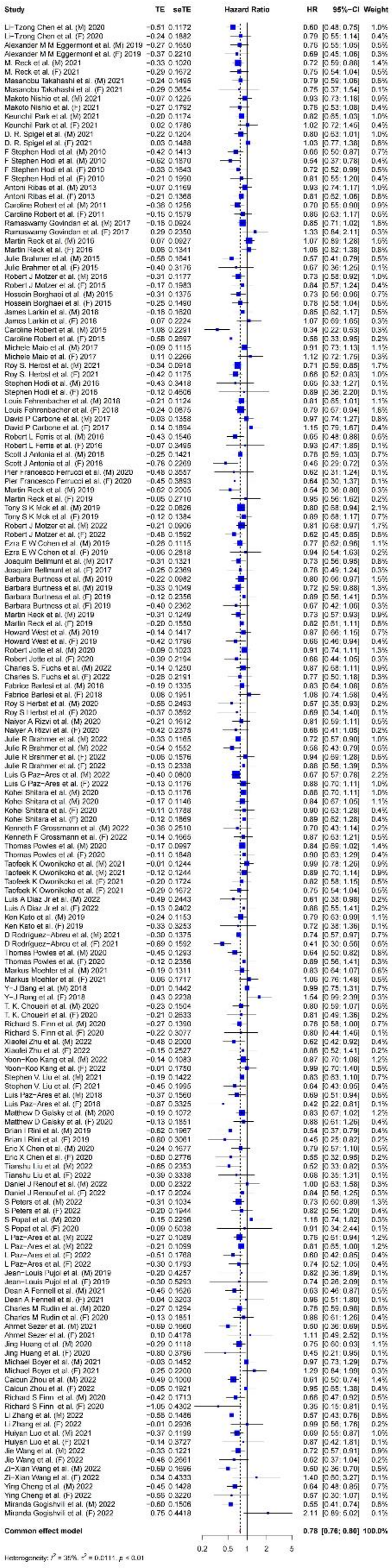

Figure S3. Subgroup analysis of hazard ratios for death in tumor patients stratified by tumor type and ICI drug type, by sex, with OS as the outcome.

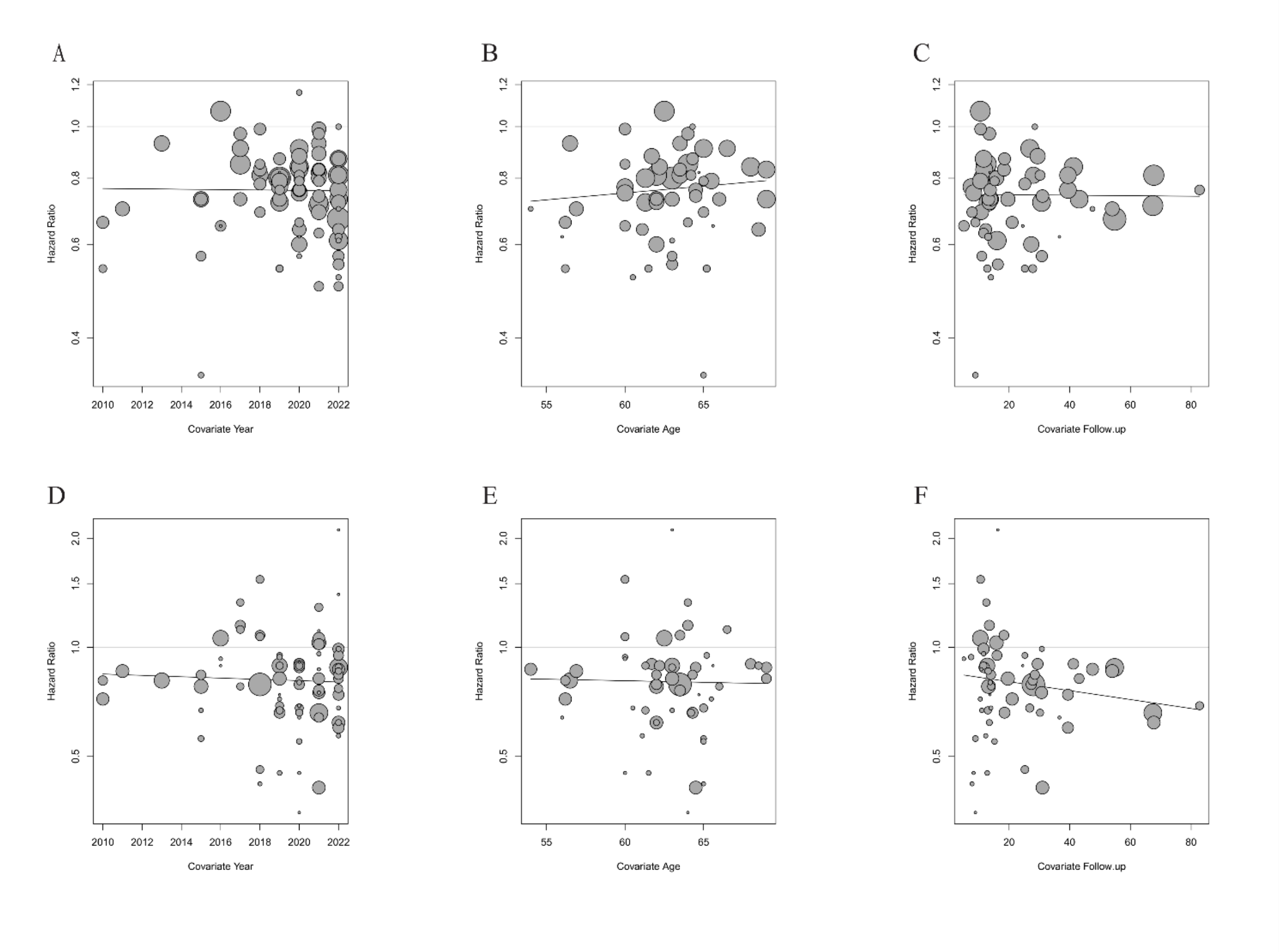

Figure S4: Meta-regression for HR of males and females, with OS as the outcome. A. Publication year (male). B. Age (male). C. Median follow up (male). D. Publication year (female). E. Age (female). F. Median follow up (female).

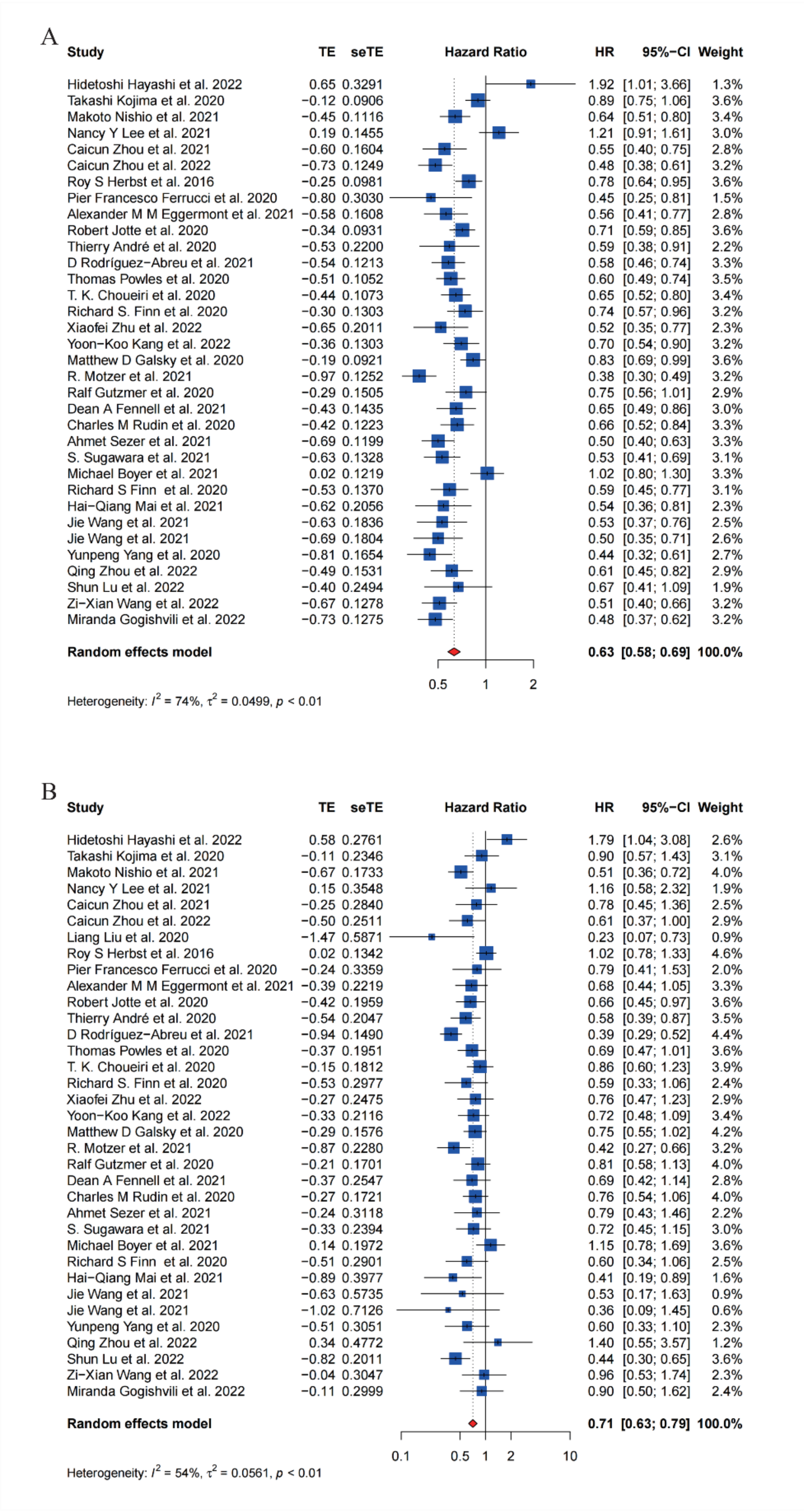

Figure S5. Hazard ratios for death in the intervention and control groups in males (A) and females (B), with PFS as the outcome.

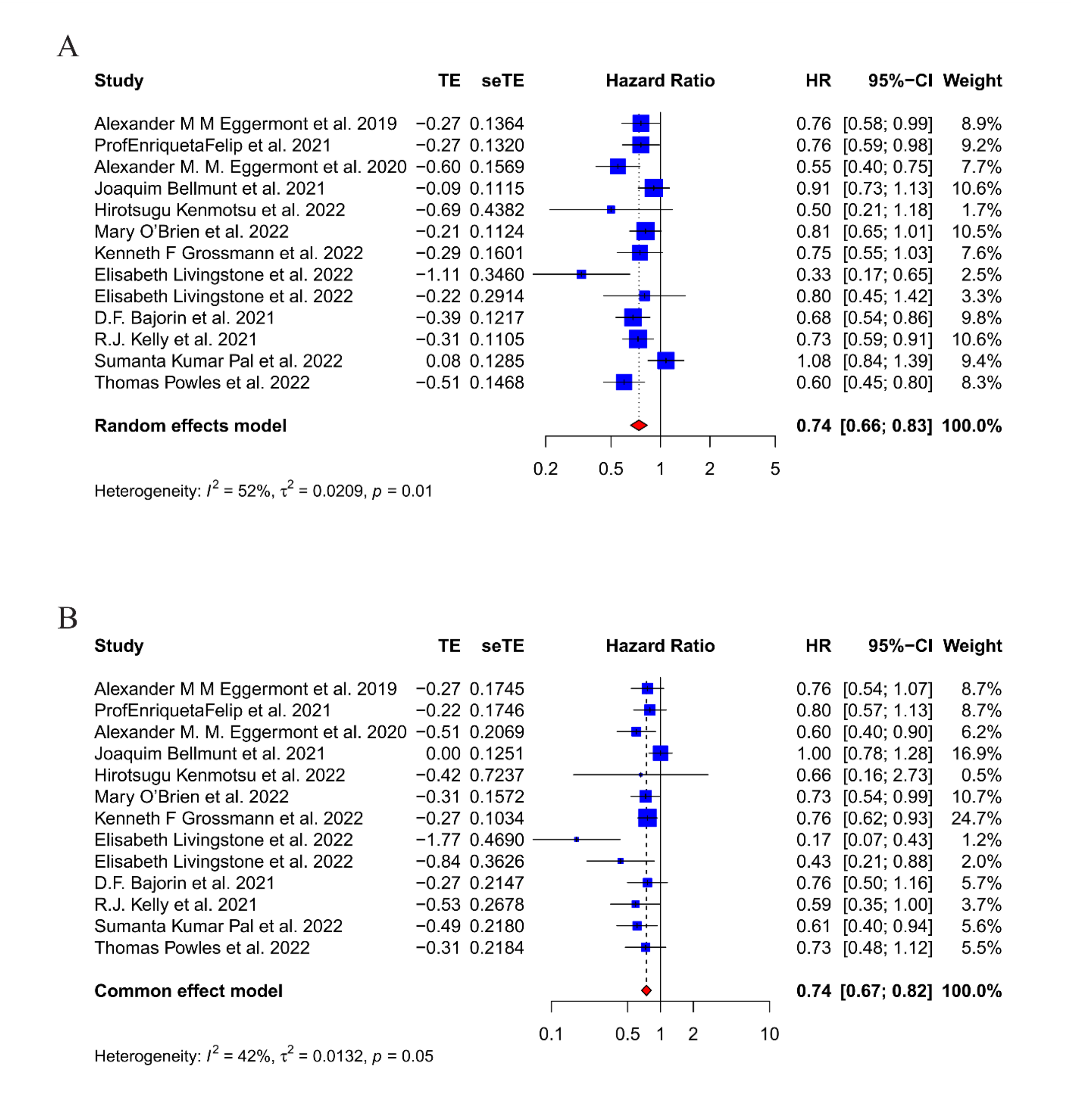

Figure S6. Hazard ratios for death in the intervention and control groups in males (A) and females (B), with RFS as the outcome.

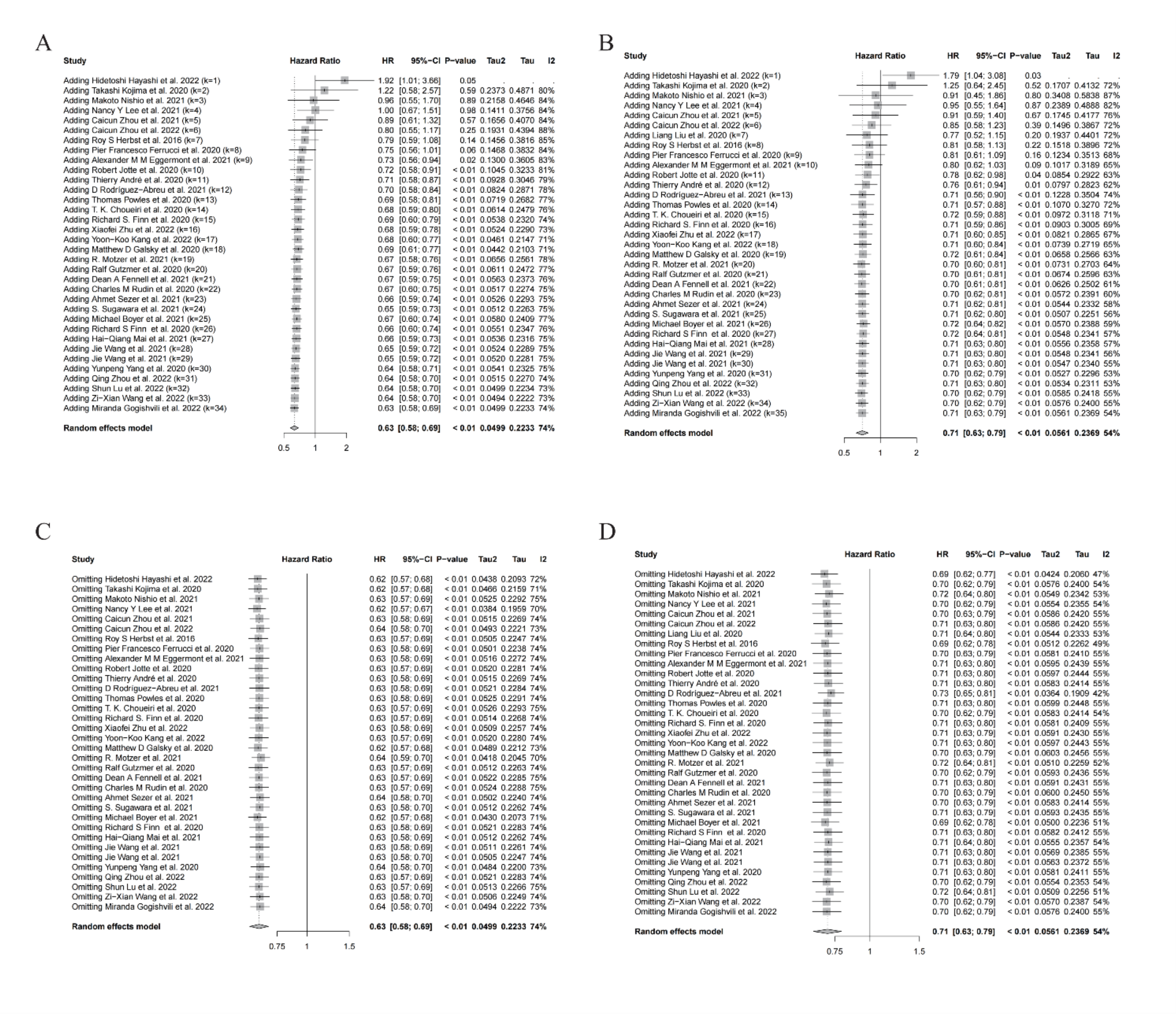

Figure S7. Cumulative meta-analysis and Sensitivity analysis for HR with PFS as the outcome. A) Cumulative meta-analysis for males by stepwise adding the included studies. B) Cumulative meta-analysis for females by stepwise adding the included studies. C) Sensitivity analysis for males by stepwise omitting the included studies. D) Sensitivity analysis for females by stepwise omitting the included studies.

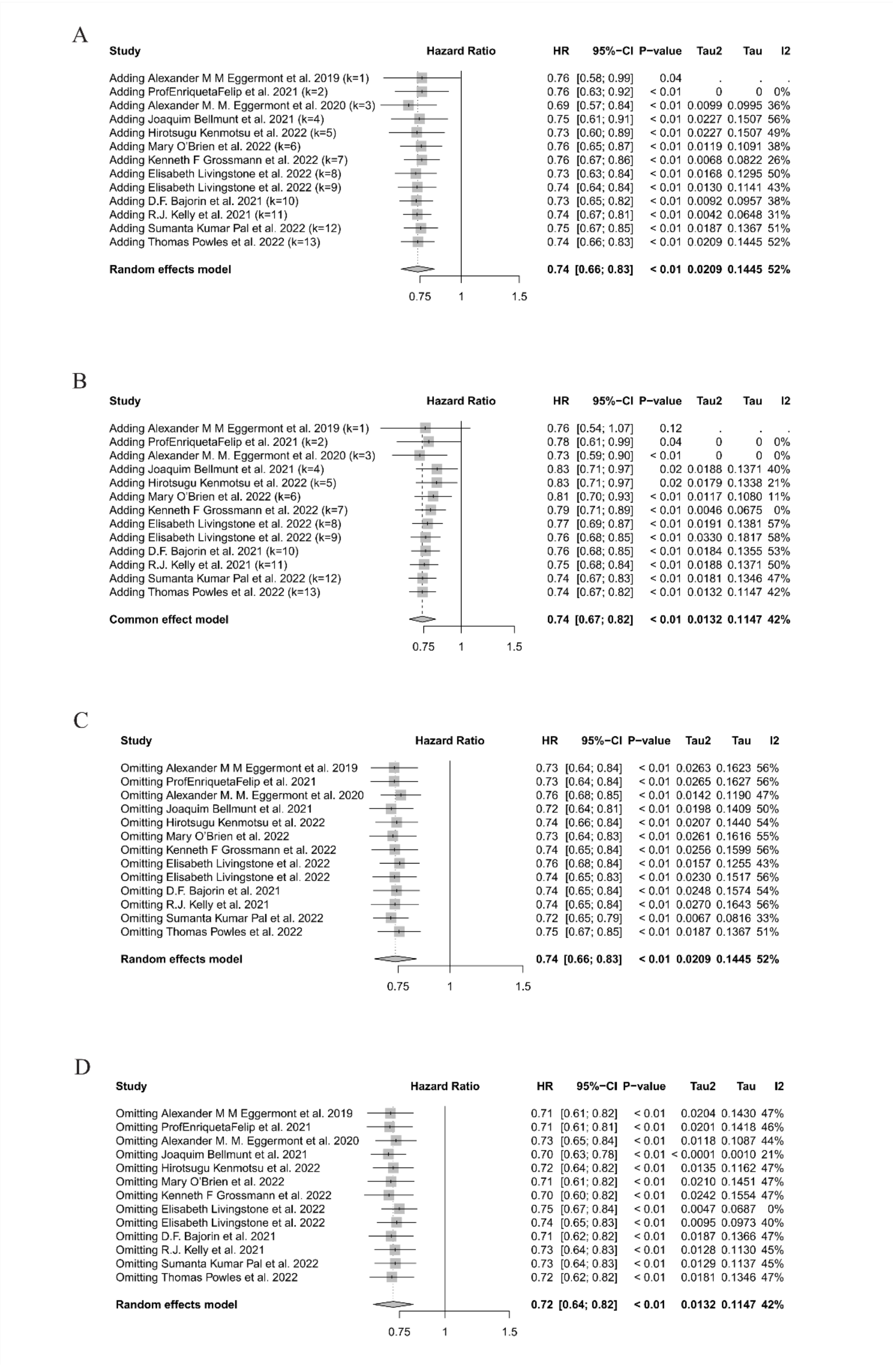

Figure S8. Cumulative meta-analysis and Sensitivity analysis for HR with RFS as the outcome. A) Cumulative meta-analysis for males by stepwise adding the included studies. B) Cumulative meta-analysis for females by stepwise adding the included studies. C) Sensitivity analysis for males by stepwise omitting the included studies. D) Sensitivity analysis for females by stepwise omitting the included studies.

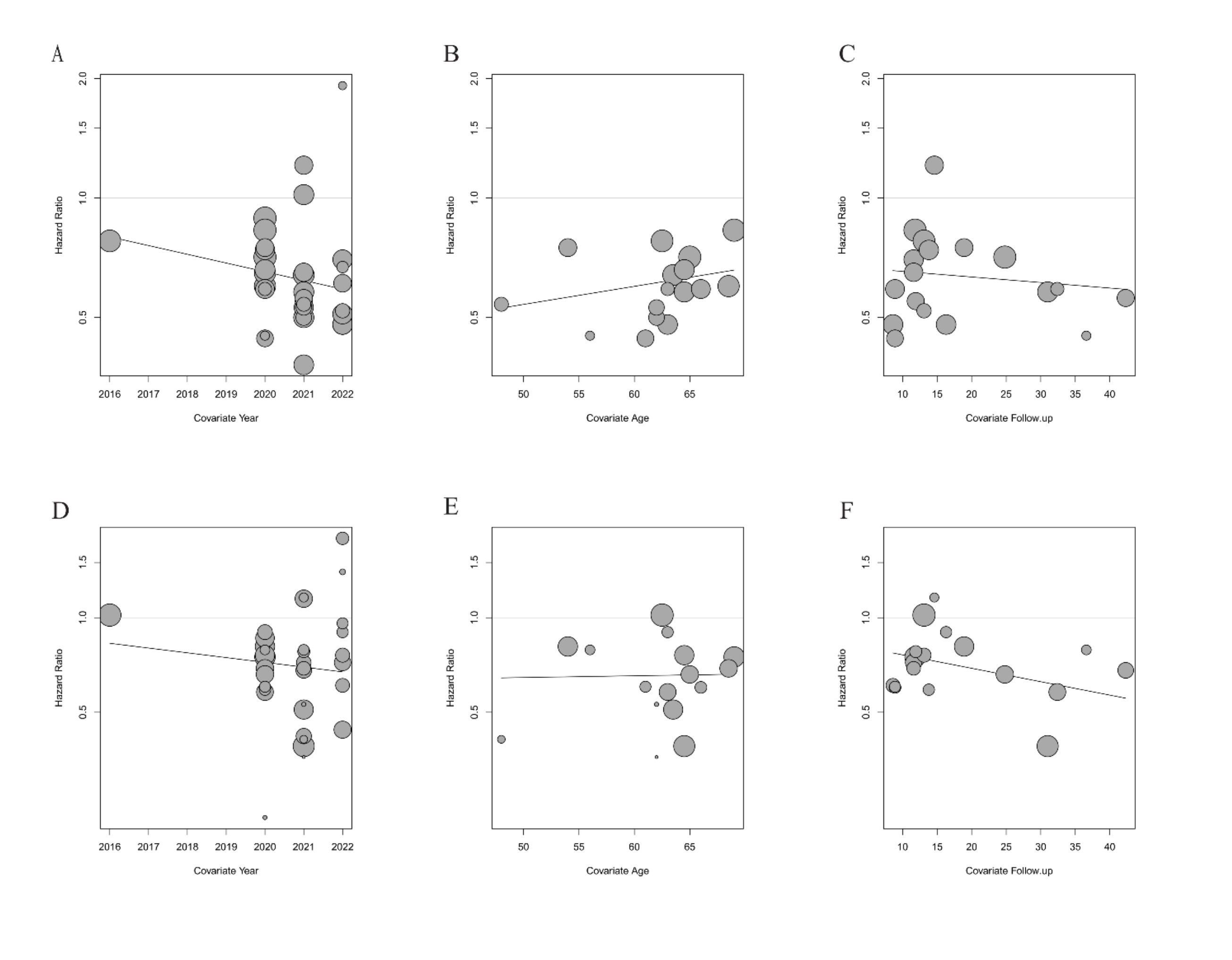

Figure S9. Meta-regression for HR of males and females, with PFS as the outcome. A) Publication year (male). B) Age (male). C) Median follow up (male). D) Publication year (female). E) Age (female). F) Median follow up (female).

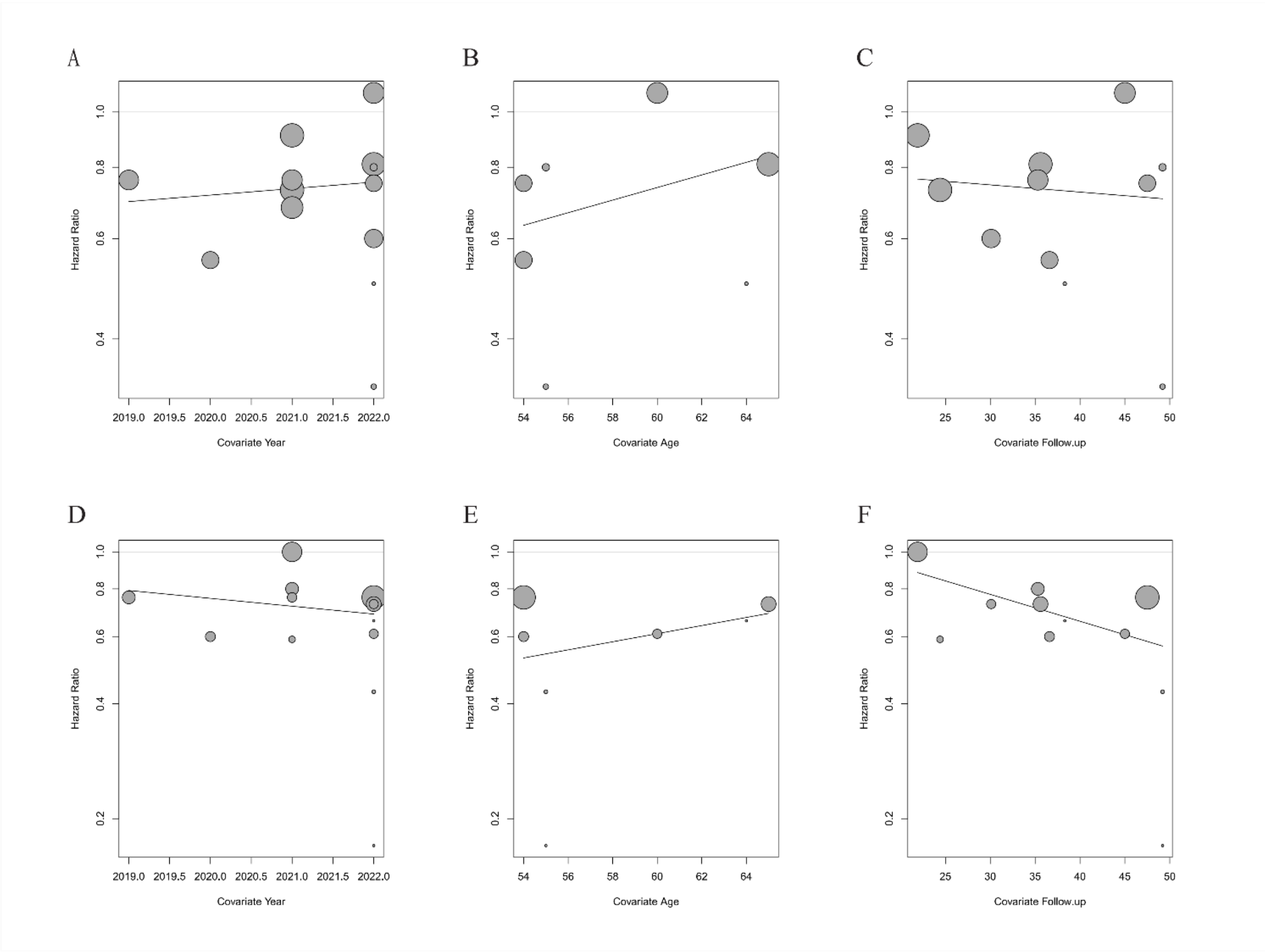

Figure S10. Meta-regression for HR of males and females, with RFS as the outcome. A) Publication year (male). B) Age (male). C) Median follow up (male). D) Publication year (female). E) Age (female). F) Median follow up (female).

1.2 Supplementary Tables

Table S1. PRISMA 2020 Checklist

Section and Topic	Item #	Checklist item	Location where item is reported
**TITLE**			
Title	1	Identify the report as a systematic review.	1
**ABSTRACT**			
Abstract	2	Provide a structured summary including, as applicable: background; objectives; data sources; study eligibility criteria, participants, and interventions; study appraisal and synthesis methods; results; limitations; conclusions and implications of key findings; systematic review registration number.	1
**INTRODUCTION**			
Rationale	3	Describe the rationale for the review in the context of existing knowledge.	2
Objectives	4	Provide an explicit statement of the objective(s) or question(s) the review addresses.	2
**METHODS**			
Eligibility criteria	5	Specify the inclusion and exclusion criteria for the review and how studies were grouped for the syntheses.	2
Information sources	6	Specify all databases, registers, websites, organisations, reference lists and other sources searched or consulted to identify studies. Specify the date when each source was last searched or consulted.	2
Search strategy	7	Present the full search strategies for all databases, registers and websites, including any filters and limits used.	Supplementary Table S2-5
Selection process	8	Specify the methods used to decide whether a study met the inclusion criteria of the review, including how many reviewers screened each record and each report retrieved, whether they worked independently, and if applicable, details of automation tools used in the process.	2-3
Data collection process	9	Specify the methods used to collect data from reports, including how many reviewers collected data from each report, whether they worked independently, any processes for obtaining or confirming data from study investigators, and if applicable, details of automation tools used in the process.	3
Data items	10a	List and define all outcomes for which data were sought. Specify whether all results that were compatible with	3 and Fig. 1

Section and Topic	Item #	Checklist item	Location where item is reported
		each outcome domain in each study were sought (e.g. for all measures, time points, analyses), and if not, the methods used to decide which results to collect.	
	10b	List and define all other variables for which data were sought (e.g. participant and intervention characteristics, funding sources). Describe any assumptions made about any missing or unclear information.	3
Study risk of bias assessment	11	Specify the methods used to assess risk of bias in the included studies, including details of the tool(s) used, how many reviewers assessed each study and whether they worked independently, and if applicable, details of automation tools used in the process.	3
Effect measures	12	Specify for each outcome the effect measure(s) (e.g. risk ratio, mean difference) used in the synthesis or presentation of results.	Table 1
Synthesis methods	13a	Describe the processes used to decide which studies were eligible for each synthesis (e.g. tabulating the study intervention characteristics and comparing against the planned groups for each synthesis (item #5)).	3 and Table 1
	13b	Describe any methods required to prepare the data for presentation or synthesis, such as handling of missing summary statistics, or data conversions.	3
	13c	Describe any methods used to tabulate or visually display results of individual studies and syntheses.	Table 1
	13d	Describe any methods used to synthesize results and provide a rationale for the choice(s). If meta-analysis was performed, describe the model(s), method(s) to identify the presence and extent of statistical heterogeneity, and software package(s) used.	3
	13e	Describe any methods used to explore possible causes of heterogeneity among study results (e.g. subgroup analysis, meta-regression).	3
	13f	Describe any sensitivity analyses conducted to assess robustness of the synthesized results.	3
Reporting bias assessment	14	Describe any methods used to assess risk of bias due to missing results in a synthesis (arising from reporting biases).	3
Certainty assessment	15	Describe any methods used to assess certainty (or confidence) in the body of evidence for an outcome.	3 and Fig. 3
**RESULTS**			
Study	16a	Describe the results of the search and selection process,	3-4 and Fig. 1

Section and Topic	Item #	Checklist item	Location where item is reported
selection		from the number of records identified in the search to the number of studies included in the review, ideally using a flow diagram.	
	16b	Cite studies that might appear to meet the inclusion criteria, but which were excluded, and explain why they were excluded.	3-4 and Fig. 1
Study characteristics	17	Cite each included study and present its characteristics.	5, Fig. 2 and Table 1
Risk of bias in studies	18	Present assessments of risk of bias for each included study.	4 and Supplementary Table 6
Results of individual studies	19	For all outcomes, present, for each study: (a) summary statistics for each group (where appropriate) and (b) an effect estimate and its precision (e.g. confidence/credible interval), ideally using structured tables or plots.	Table 1
Results of syntheses	20a	For each synthesis, briefly summarise the characteristics and risk of bias among contributing studies.	21-24 and Fig. 3-4
	20b	Present results of all statistical syntheses conducted. If meta-analysis was done, present for each the summary estimate and its precision (e.g. confidence/credible interval) and measures of statistical heterogeneity. If comparing groups, describe the direction of the effect.	21-24 and Fig. 3
	20c	Present results of all investigations of possible causes of heterogeneity among study results.	Fig. 3
	20d	Present results of all sensitivity analyses conducted to assess the robustness of the synthesized results.	Supplementary Figure 1-2, 21-24
Reporting biases	21	Present assessments of risk of bias due to missing results (arising from reporting biases) for each synthesis assessed.	Supplementary Figure 1-2, 21-24
Certainty of evidence	22	Present assessments of certainty (or confidence) in the body of evidence for each outcome assessed.	9-11 and Fig. 3
**DISCUSSION**			
Discussion	23a	Provide a general interpretation of the results in the context of other evidence.	24-26
	23b	Discuss any limitations of the evidence included in the review.	25-26
	23c	Discuss any limitations of the review processes used.	25-26
	23d	Discuss implications of the results for practice, policy,	25-26

Section and Topic	Item #	Checklist item	Location where item is reported
		and future research.	
**OTHER INFORMATION**			
Registration and protocol	24a	Provide registration information for the review, including register name and registration number, or state that the review was not registered.	2-3
	24b	Indicate where the review protocol can be accessed, or state that a protocol was not prepared.	2-3
	24c	Describe and explain any amendments to information provided at registration or in the protocol.	2-3
Support	25	Describe sources of financial or non-financial support for the review, and the role of the funders or sponsors in the review.	26
Competing interests	26	Declare any competing interests of review authors.	27
Availability of data, code and other materials	27	Report which of the following are publicly available and where they can be found: template data collection forms; data extracted from included studies; data used for all analyses; analytic code; any other materials used in the review.	Methods and Supplementary

From: Page, M.J.; McKenzie, J.E.; Bossuyt, P.M.; Boutron, I.; Hoffmann, T.C.; Mulrow, C.D.; Shamseer, L.; Tetzlaff, J.M.; Akl, E.A.; Brennan, S.E.; et al. The PRISMA 2020 statement: An updated guideline for reporting systematic reviews. Rev. Esp. Cardiol. 2021, 74, 790–799. https://doi.org/10.1016/j.rec.2021.07.010

Table S2. Details of Search Strategy to Retrieve the Studies using PubMed (Medline). Date of Search: 11/28/2023.

#	Search Terms	Hits
#1	Search: (((((((((((((((((Immune Checkpoint Inhibitors[MeSH Terms]) OR (Immune Checkpoint Inhibitors[Title/Abstract])) OR (immunotherapy[Title/Abstract])) OR (Immune Checkpoint Proteins[MeSH Terms])) OR (Immune Checkpoint Proteins[Title/Abstract])) OR (CTLA-4[Title/Abstract])) OR (cytotoxic T-lymphocyte-associated protein 4[Title/Abstract])) OR (PD-1[Title/Abstract])) OR (programmed death receptor 1[Title/Abstract])) OR (PD-L1[Title/Abstract])) OR (programmed cell death 1 ligand 1 protein[Title/Abstract])) OR (atezolizumab[Title/Abstract])) OR (ipilimumab[Title/Abstract])) OR (tremelimumab[Title/Abstract])) OR (nivolumab[Title/Abstract])) OR (pembrolizumab[Title/Abstract])) OR (LAG-3[Title/Abstract])) OR (TIM-3[Title/Abstract])) OR (TIGIT[Title/Abstract])	189,733

#2	Search: ((((((Randomized Controlled Trial[Publication Type]) OR (Randomized Controlled Trial[Title/Abstract])) OR (Clinical Trials as Topic[MeSH Terms])) OR (controlled clinical trial[Publication Type])) OR (randomized[Title/Abstract])) OR (placebo[Title/Abstract])) OR (drug therapy[MeSH Terms]) OR (randomly[Title/Abstract])	2,859,642
#3	Search: (animals[MeSH Terms]) NOT (humans[MeSH Terms])	5,172,521
#4	Search: #2 NOT #3	2,476,546
#5	Search: ((((((((((((("Neoplasms"[Mesh]) OR (Tumor[Title/Abstract])) OR (Neoplasm[Title/Abstract])) OR (Tumors[Title/Abstract])) OR (Neoplasia[Title/Abstract])) OR (Neoplasias[Title/Abstract])) OR (Cancer[Title/Abstract])) OR (Cancers[Title/Abstract])) OR (Malignant Neoplasm[Title/Abstract])) OR (Malignancy[Title/Abstract])) OR (Malignancies[Title/Abstract])) OR (Malignant Neoplasms[Title/Abstract])) OR (Neoplasm, Malignant[Title/Abstract])) OR (Neoplasms, Malignant[Title/Abstract])	4,971,333
**#6**	**#1 AND #4 AND #5**	**22,347**

Table S3. Details of Search Strategy to Retrieve the Studies using Embase. Date of Search: 11/28/2023.

#	Search Terms	Hits
#1	(Immune Checkpoint Inhibitors or immunotherapy or Immune Checkpoint Proteins or cytotoxic T-lymphocyte-associated protein 4 or programmed death 1 receptor or programmed death 1 ligand 1 or CTLA-4 or PD-1 or PD-L1 or atezolizumab or ipilimumab or tremelimumab or nivolumab or pembrolizumab or LAG-3 or TIM-3 or TIGIT).ab,kw,ti.	284309
#2	(Randomized Controlled Trial or controlled clinical trial or randomized or placebo or randomly or drug therapy).ab,kw,ti.	1654454

#3	(neoplasm or malignant neoplasm or cancer or tumor).ab,kw,ti.	4194 671
**#4**	**#1 AND #2 AND #3**	**1118 5**

Table S4. Details of Search Strategy to Retrieve the Studies using Cochrane. Date of Search: 11/28/2023.

#	Search Terms	Hits
#1	(Immune Checkpoint Inhibitors):ti,ab,kw or (immunotherapy):ti,ab,kw or (Immune Checkpoint Proteins):ti,ab,kw or (cytotoxic T-lymphocyte-associated protein 4):ti,ab,kw or (programmed death 1 receptor):ti,ab,kw or (programmed death 1 ligand 1):ti,ab,kw or (CTLA-4):ti,ab,kw or (PD-1):ti,ab,kw or (PD-L1):ti,ab,kw or (atezolizumab):ti,ab,kw or (ipilimumab):ti,ab,kw or (tremelimumab):ti,ab,kw or (nivolumab):ti,ab,kw or (pembrolizumab):ti,ab,kw or (LAG-3):ti,ab,kw or (TIM-3):ti,ab,kw or (TIGIT):ti,ab,kw	2100 5
#2	(Randomized Controlled Trial):ti,ab,kw or (Clinical Trials as Topic):ti,ab,kw or (controlled clinical trial):ti,ab,kw or (randomized):ti,ab,kw or (placebo):ti,ab,kw or (randomly):ti,ab,kw or (drug therapy):ti,ab,kw	1461 979
#3	(Neoplasms):ti,ab,kw or (Tumor):ti,ab,kw or (Neoplasm):ti,ab,kw or (Tumors):ti,ab,kw or (Neoplasia):ti,ab,kw or (Neoplasias):ti,ab,kw or (Cancer):ti,ab,kw or (Cancers):ti,ab,kw or (Malignant Neoplasm):ti,ab,kw or (Malignancy):ti,ab,kw or (Malignancies):ti,ab,kw or (Malignant Neoplasms):ti,ab,kw or (Neoplasm, Malignant):ti,ab,kw or (Neoplasms, Malignant):ti,ab,kw	2580 13
**#4**	**#1 AND #2 AND #3**	**1232 6**

Table S5: Details of Search Strategy to Retrieve the Studies using Web of Science. Date of Search: 11/28/2023.

#	Search Terms	Hits
#1	TS = ("Immune Checkpoint Inhibitors" OR "immunotherapy" OR "Immune Checkpoint Proteins" OR "cytotoxic T-lymphocyte-associated protein 4" OR "programmed death 1 receptor" OR "programmed death 1 ligand 1" OR "CTLA-4" OR "PD-1" OR "PD-L1" OR "atezolizumab" OR "ipilimumab" OR "tremelimumab" OR "nivolumab" OR "pembrolizumab" OR "LAG-3" OR "TIM-3" OR "TIGIT")	360,154
#2	TS = ("Randomized Controlled Trial" OR "controlled clinical trial" OR "randomized" OR "placebo" OR "randomly" OR "drug therapy")	5,896,685
#3	TS = ("neoplasm" OR "malignant neoplasm")	947,212
**#4**	**#1 AND #2 AND #3**	**17,198**

Table S6. Jadad quality score of included studies.

Study (year)	Randomization	Concealment of allocation	Blinding	Withdrawals and dropouts	Total
Improved survival with ipilimumab in patients with metastatic melanoma (2010)	1	1	2	1	5
Ipilimumab plus dacarbazine for previously untreated metastatic melanoma (2011)	1	1	2	1	5

Phase III randomized clinical trial comparing tremelimumab with standard-of-care chemotherapy in patients with advanced melanoma (2013)	2	1	0	1	4
Nivolumab versus Docetaxel in Advanced Nonsquamous Non-Small-Cell Lung Cancer (2015)	2	1	0	1	4
Nivolumab versus Docetaxel in Advanced Squamous-Cell Non-Small-Cell Lung Cancer (2015)	2	1	0	1	4
Nivolumab versus Everolimus in Advanced Renal-Cell Carcinoma (2015)	2	1	0	1	4
Nivolumab in previously untreated melanoma without BRAF mutation (2015)	1	1	2	1	5
Nivolumab for Recurrent Squamous-Cell Carcinoma of the Head and Neck (2016)	2	1	0	1	4
Pembrolizumab versus docetaxel for previously treated, PD-L1-positive, advanced non-small-cell lung cancer (KEYNOTE-010): a randomised controlled trial (2016)	2	2	0	1	5
Combined nivolumab and ipilimumab versus ipilimumab alone in patients with advanced melanoma: 2-year overall survival outcomes in a multicentre, randomised, controlled, phase 2 trial (2016)	2	2	2	1	7
Phase III Randomized Trial of Ipilimumab Plus Etoposide and Platinum Versus Placebo Plus Etoposide and Platinum in Extensive-Stage Small-Cell Lung Cancer (2016)	2	2	2	1	7
Pembrolizumab as Second-Line Therapy for Advanced Urothelial Carcinoma (2017)	2	1	0	1	4
First-Line Nivolumab in Stage IV or Recurrent Non-Small-Cell Lung Cancer (2017)	2	1	0	1	4

Phase III Trial of Ipilimumab Combined With Paclitaxel and Carboplatin in Advanced Squamous Non-Small-Cell Lung Cancer (2017)	2	1	2	1	6
Tremelimumab as second-line or third-line treatment in relapsed malignant mesothelioma (DETERMINE): a multicentre, international, randomised, double-blind, placebo-controlled phase 2b trial (2017)	2	2	2	1	7
Overall Survival with Durvalumab after Chemoradiotherapy in Stage III NSCLC (2018)	1	1	2	1	5
Phase III, randomised trial of avelumab versus physician's choice of chemotherapy as third-line treatment of patients with advanced gastric or gastro-oesophageal junction cancer: primary analysis of JAVELIN Gastric 300 (2018)	1	1	2	1	5
Avelumab versus docetaxel in patients with platinum-treated advanced non-small-cell lung cancer (JAVELIN Lung 200): an open-label, randomised, phase 3 study (2018)	2	2	0	1	5
Updated Efficacy Analysis Including Secondary Population Results for OAK: A Randomized Phase III Study of Atezolizumab versus Docetaxel in Patients with Previously Treated Advanced Non-Small Cell Lung Cancer (2018)	2	2	0	1	5
Overall Survival in Patients With Advanced Melanoma Who Received Nivolumab Versus Investigator's Choice Chemotherapy in CheckMate 037: A Randomized, Controlled, Open-Label Phase III Trial (2018)	2	2	0	1	5
Pembrolizumab plus Chemotherapy for Squamous Non-Small-Cell Lung Cancer (2018)	2	2	2	1	7

Pembrolizumab alone or with chemotherapy versus cetuximab with chemotherapy for recurrent or metastatic squamous cell carcinoma of the head and neck (KEYNOTE-048): a randomised, open-label, phase 3 study (2019)	2	2	0	1	5
Pembrolizumab versus methotrexate, docetaxel, or cetuximab for recurrent or metastatic head-and-neck squamous cell carcinoma (KEYNOTE-040): a randomised, open-label, phase 3 study (2019)	2	2	0	1	5
Adjuvant ipilimumab versus placebo after complete resection of stage III melanoma: long-term follow-up results of the European Organisation for Research and Treatment of Cancer 18071 double-blind phase 3 randomised trial (2019)	1	2	2	0	5
Nivolumab versus chemotherapy in patients with advanced oesophageal squamous cell carcinoma refractory or intolerant to previous chemotherapy (ATTRACTION-3): a multicentre, randomised, open-label, phase 3 trial (2019)	2	2	0	1	5
Pembrolizumab versus chemotherapy for previously untreated, PD-L1-expressing, locally advanced or metastatic non-small-cell lung cancer (KEYNOTE-042): a randomised, open-label, controlled, phase 3 trial (2019)	2	2	0	1	5
A Randomized Non-Comparative Phase II Study of Anti-Programmed Cell Death-Ligand 1 Atezolizumab or Chemotherapy as Second-Line Therapy in Patients With Small Cell Lung Cancer: Results From the IFCT-1603 Trial (2019)	2	1	0	1	4
Atezolizumab plus bevacizumab and chemotherapy in non-small-cell lung cancer (IMpower150): key subgroup analyses of patients with EGFR mutations or baseline liver	2	2	0	1	5

metastases in a randomised, open-label phase 3 trial (2019)					
Updated Analysis of KEYNOTE-024: Pembrolizumab Versus Platinum-Based Chemotherapy for Advanced Non-Small-Cell Lung Cancer With PD-L1 Tumor Proportion Score of 50% or Greater (2019)	2	1	0	1	4
Pembrolizumab plus Axitinib versus Sunitinib for Advanced Renal-Cell Carcinoma (2019)	2	1	0	1	4
Atezolizumab in combination with carboplatin plus nab-paclitaxel chemotherapy compared with chemotherapy alone as first-line treatment for metastatic non-squamous non-small-cell lung cancer (IMpower130): a multicentre, randomised, open-label, phase 3 trial (2019)	2	2	0	1	5
Pembrolizumab in Microsatellite-Instability-High Advanced Colorectal Cancer (2020)	2	1	0	1	4
Effect of Combined Immune Checkpoint Inhibition vs Best Supportive Care Alone in Patients With Advanced Colorectal Cancer: The Canadian Cancer Trials Group CO.26 Study (2020)	2	1	0	1	4
A phase 3 study of nivolumab in previously treated advanced gastric or gastroesophageal junction cancer (ATTRACTION-2): 2-year update data (2020)	2	2	1	1	6
Updated efficacy results from the JAVELIN Renal 101 trial: first-line avelumab plus axitinib versus sunitinib in patients with advanced renal cell carcinoma (2020)	2	1	0	1	4
Association Between Immune-Related Adverse Events and Recurrence-Free Survival Among Patients With Stage III Melanoma Randomized to Receive Pembrolizumab or	1	2	2	1	6

Placebo: A Secondary Analysis of a Randomized Clinical Trial (2020)					
KEYNOTE-022 part 3: a randomized, double-blind, phase 2 study of pembrolizumab, dabrafenib, and trametinib in BRAF-mutant melanoma (2020)	2	2	2	0	6
Atezolizumab plus Bevacizumab in Unresectable Hepatocellular Carcinoma (2020)	2	2	0	0	4
Pembrolizumab As Second-Line Therapy in Patients With Advanced Hepatocellular Carcinoma in KEYNOTE-240: A Randomized, Double-Blind, Phase III Trial (2020)	2	2	2	1	7
Atezolizumab with or without chemotherapy in metastatic urothelial cancer (IMvigor130): a multicentre, randomised, placebo-controlled phase 3 trial (2020)	2	2	1	1	6
Atezolizumab, vemurafenib, and cobimetinib as first-line treatment for unresectable advanced BRAF (V600) mutation-positive melanoma (IMspire150): primary analysis of the randomised, double-blind, placebo-controlled, phase 3 trial (2020)	2	2	2	1	7
Atezolizumab for first-line treatment of PD-L1-selected patients with NSCLC (2020)	2	1	0	1	4
Camrelizumab versus investigator's choice of chemotherapy as second-line therapy for advanced or metastatic oesophageal squamous cell carcinoma (ESCORT): a multicentre, randomised, open-label, phase 3 study (2020)	2	2	0	1	5
Atezolizumab in Combination With Carboplatin and Nab-Paclitaxel in Advanced Squamous NSCLC (IMpower131): Results From a Randomized Phase III Trial (2020)	2	1	0	1	4

Randomized Phase III KEYNOTE-181 Study of Pembrolizumab Versus Chemotherapy in Advanced Esophageal Cancer (2020)	2	1	0	1	4
A multicentre randomised phase III trial comparing pembrolizumab versus single-agent chemotherapy for advanced pre-treated malignant pleural mesothelioma: the European Thoracic Oncology Platform (ETOP 9-15) PROMISE-meso trial (2020)	2	2	0	1	5
Avelumab Maintenance Therapy for Advanced or Metastatic Urothelial Carcinoma (2020)	2	1	0	1	4
Durvalumab alone and durvalumab plus tremelimumab versus chemotherapy in previously untreated patients with unresectable, locally advanced or metastatic urothelial carcinoma (DANUBE): a randomised, open-label, multicentre, phase 3 trial (2020)	2	2	0	1	5
Durvalumab With or Without Tremelimumab vs Standard Chemotherapy in First-line Treatment of Metastatic Non-Small Cell Lung Cancer: The MYSTIC Phase 3 Randomized Clinical Trial (2020)	2	1	0	1	4
Pembrolizumab or Placebo Plus Etoposide and Platinum as First-Line Therapy for Extensive-Stage Small-Cell Lung Cancer: Randomized, Double-Blind, Phase III KEYNOTE-604 Study (2020)	2	2	2	1	7
Efficacy and Safety of Pembrolizumab or Pembrolizumab Plus Chemotherapy vs Chemotherapy Alone for Patients With First-line, Advanced Gastric Cancer: The KEYNOTE-062 Phase 3 Randomized Clinical Trial (2020)	2	2	1	1	6
Efficacy and Safety of Sintilimab Plus Pemetrexed and Platinum as First-Line Treatment for Locally Advanced or Metastatic	2	2	2	0	6

Nonsquamous NSCLC: a Randomized, Double-Blind, Phase 3 Study (Oncology pRogram by InnovENT anti-PD-1-11) (2020)					
Adjuvant Nivolumab versus Placebo in Muscle-Invasive Urothelial Carcinoma (2021)	2	2	1	1	6
Adjuvant atezolizumab versus observation in muscle-invasive urothelial carcinoma (IMvigor010): a multicentre, open-label, randomised, phase 3 trial (2021)	2	2	0	1	5
Pembrolizumab Plus Ipilimumab or Placebo for Metastatic Non-Small-Cell Lung Cancer With PD-L1 Tumor Proportion Score >= 50%: Randomized, Double-Blind Phase III KEYNOTE-598 Study (2021)	2	2	2	1	7
Adjuvant pembrolizumab versus placebo in resected stage III melanoma (EORTC 1325-MG/KEYNOTE-054): distant metastasis-free survival results from a double-blind, randomised, controlled, phase 3 trial (2021)	2	2	2	0	6
Adjuvant atezolizumab after adjuvant chemotherapy in resected stage IB-IIIA non-small-cell lung cancer (IMpower010): a randomised, multicentre, open-label, phase 3 trial (2021)	2	2	0	1	5
Nivolumab versus placebo in patients with relapsed malignant mesothelioma (CONFIRM): a multicentre, double-blind, randomised, phase 3 trial (2021)	2	2	2	1	7
Five Year Survival Update From KEYNOTE-010: Pembrolizumab Versus Docetaxel for Previously Treated, Programmed Death-Ligand 1-Positive Advanced NSCLC (2021)	2	2	0	0	4
Adjuvant Nivolumab in Resected Esophageal or Gastroesophageal Junction Cancer (2021)	2	2	2	1	7

Avelumab plus standard-of-care chemoradiotherapy versus chemoradiotherapy alone in patients with locally advanced squamous cell carcinoma of the head and neck: a randomised, double-blind, placebo-controlled, multicentre, phase 3 trial (2021)	2	2	2	1	7
Updated Overall Survival and PD-L1 Subgroup Analysis of Patients With Extensive-Stage Small-Cell Lung Cancer Treated With Atezolizumab, Carboplatin, and Etoposide (IMpower133) (2021)	1	1	2	1	5
Effect of Camrelizumab vs Placebo Added to Chemotherapy on Survival and Progression-Free Survival in Patients With Advanced or Metastatic Esophageal Squamous Cell Carcinoma: The ESCORT-1st Randomized Clinical Trial (2021)	2	2	2	1	7
Toripalimab or placebo plus chemotherapy as first-line treatment in advanced nasopharyngeal carcinoma: a multicenter randomized phase 3 trial (2021)	2	2	2	0	6
Phase III Trial of Avelumab Maintenance After First-Line Induction Chemotherapy Versus Continuation of Chemotherapy in Patients With Gastric Cancers: Results From JAVELIN Gastric 100 (2021)	2	1	0	1	4
Lenvatinib plus Pembrolizumab or Everolimus for Advanced Renal Cell Carcinoma (2021)	2	1	0	1	4
Atezolizumab Plus Chemotherapy for First-Line Treatment of Nonsquamous NSCLC: Results From the Randomized Phase 3 IMpower132 Trial (2021)	2	2	0	1	5
Nivolumab and Ipilimumab as Maintenance Therapy in Extensive-Disease Small-Cell Lung Cancer: CheckMate 451 (2021)	1	1	2	0	4

Avelumab Versus Docetaxel in Patients With Platinum-Treated Advanced NSCLC: 2-Year Follow-Up From the JAVELIN Lung 200 Phase 3 Trial (2021)	2	1	0	1	4
First-line nivolumab plus ipilimumab with two cycles of chemotherapy versus chemotherapy alone (four cycles) in advanced non-small-cell lung cancer: CheckMate 9LA 2-year update (2021)	2	1	0	1	4
Pemetrexed plus platinum with or without pembrolizumab in patients with previously untreated metastatic nonsquamous NSCLC: protocol-specified final analysis from KEYNOTE-189 (2021)	2	2	2	0	6
Cemiplimab monotherapy for first-line treatment of advanced non-small-cell lung cancer with PD-L1 of at least 50%: a multicentre, open-label, global, phase 3, randomised, controlled trial (2021)	2	2	0	0	4
Second-line nivolumab in relapsed small-cell lung cancer: CheckMate 331 (☆) (2021)	2	2	0	1	5
Nivolumab with carboplatin, paclitaxel, and bevacizumab for first-line treatment of advanced nonsquamous non-small-cell lung cancer (2021)	1	1	2	1	5
Nivolumab versus chemotherapy in Japanese patients with advanced esophageal squamous cell carcinoma: a subgroup analysis of a multicenter, randomized, open-label, phase 3 trial (ATTRACTION-3) (2021)	2	2	0	0	4
Tislelizumab Plus Chemotherapy vs Chemotherapy Alone as First-line Treatment for Advanced Squamous Non-Small-Cell Lung Cancer: A Phase 3 Randomized Clinical Trial (2021)	2	2	0	1	5

Camrelizumab plus carboplatin and pemetrexed versus chemotherapy alone in chemotherapy-naive patients with advanced non-squamous non-small-cell lung cancer (CameL): a randomised, open-label, multicentre, phase 3 trial (2021)	2	2	0	1	5
Effect of First-Line Serplulimab vs Placebo Added to Chemotherapy on Survival in Patients With Extensive-Stage Small Cell Lung Cancer: The ASTRUM-005 Randomized Clinical Trial (2022)	2	2	2	1	7
Pembrolizumab versus chemotherapy for microsatellite instability-high or mismatch repair-deficient metastatic colorectal cancer (KEYNOTE-177): final analysis of a randomised, open-label, phase 3 study (2022)	2	2	0	1	5
Pembrolizumab versus paclitaxel for previously treated PD-L1-positive advanced gastric or gastroesophageal junction cancer: 2-year update of the randomized phase 3 KEYNOTE-061 trial (2022)	2	2	0	1	5
Cemiplimab plus chemotherapy versus chemotherapy alone in non-small cell lung cancer: a randomized, controlled, double-blind phase 3 trial (2022)	2	2	2	1	7
Adjuvant Pembrolizumab versus IFNα2b or Ipilimumab in Resected High-Risk Melanoma (2022)	2	1	0	1	4
A Randomized Phase II Study Comparing Nivolumab with Carboplatin-Pemetrexed for EGFR-Mutated NSCLC with Resistance to EGFR Tyrosine Kinase Inhibitors (WJOG8515L) (2022)	2	1	0	1	4
Nivolumab plus chemotherapy versus placebo plus chemotherapy in patients with HER2-negative, untreated, unresectable advanced or recurrent gastric or gastro-oesophageal	2	2	2	1	7

junction cancer (ATTRACTION-4): a randomised, multicentre, double-blind, placebo-controlled, phase 3 trial (2022)					
Adjuvant atezolizumab in Japanese patients with resected stage IB-IIIA non-small cell lung cancer (IMpower010) (2022)	2	2	0	1	5
First-line nivolumab plus chemotherapy vs chemotherapy in patients with advanced gastric, gastroesophageal junction, and esophageal adenocarcinoma: CheckMate 649 Chinese subgroup analysis (2022)	2	2	0	1	5
Adjuvant nivolumab plus ipilimumab or nivolumab alone versus placebo in patients with resected stage IV melanoma with no evidence of disease (IMMUNED): final results of a randomised, double-blind, phase 2 trial (2022)	2	2	2	0	6
Sintilimab plus bevacizumab biosimilar IBI305 and chemotherapy for patients with EGFR-mutated non-squamous non-small-cell lung cancer who progressed on EGFR tyrosine-kinase inhibitor therapy (ORIENT-31): first interim results from a randomised, double-blind, multicentre, phase 3 trial (2022)	2	2	2	1	7
Biomarker analysis from CheckMate 214: nivolumab plus ipilimumab versus sunitinib in renal cell carcinoma (2022)	2	1	0	1	4
Pembrolizumab versus placebo as adjuvant therapy for completely resected stage IB-IIIA non-small-cell lung cancer (PEARLS/KEYNOTE-091): an interim analysis of a randomised, triple-blind, phase 3 trial (2022)	2	2	2	1	7
Adjuvant atezolizumab versus placebo for patients with renal cell carcinoma at increased risk of recurrence following resection (IMmotion010): a multicentre, randomised, double-blind, phase 3 trial (2022)	2	2	2	1	7

Durvalumab, with or without tremelimumab, plus platinum-etoposide in first-line treatment of extensive-stage small-cell lung cancer: 3-year overall survival update from CASPIAN (2022)	2	2	0	1	5
First-Line Nivolumab Plus Ipilimumab in Advanced NSCLC: 4-Year Outcomes From the Randomized, Open-Label, Phase 3 CheckMate 227 Part 1 Trial (2022)	2	1	0	1	4
First-line nivolumab plus ipilimumab versus chemotherapy in patients with unresectable malignant pleural mesothelioma: 3-year outcomes from CheckMate 743 (2022)	2	1	0	1	4
Pembrolizumab versus placebo as post-nephrectomy adjuvant therapy for clear cell renal cell carcinoma (KEYNOTE-564): 30-month follow-up analysis of a multicentre, randomised, double-blind, placebo-controlled, phase 3 trial (2022)	2	2	2	1	7
The CCTG PA.7 phase II trial of gemcitabine and nab-paclitaxel with or without durvalumab and tremelimumab as initial therapy in metastatic pancreatic ductal adenocarcinoma (2022)	1	2	0	1	4
Adebrelimab or placebo plus carboplatin and etoposide as first-line treatment for extensive-stage small-cell lung cancer (CAPSTONE-1): a multicentre, randomised, double-blind, placebo-controlled, phase 3 trial (2022)	2	2	2	1	7
Toripalimab plus chemotherapy in treatment-naïve, advanced esophageal squamous cell carcinoma (JUPITER-06): A multi-center phase 3 trial (2022)	2	2	2	1	7
Final overall survival data of sintilimab plus pemetrexed and platinum as First-Line treatment for locally advanced or metastatic	1	1	2	0	4

nonsquamous NSCLC in the Phase 3 ORIENT-11 study (2022)					
Tislelizumab Versus Docetaxel in Patients With Previously Treated Advanced NSCLC (RATIONALE-303): A Phase 3, Open-Label, Randomized Controlled Trial (2022)	2	2	0	1	5
Sugemalimab versus placebo, in combination with platinum-based chemotherapy, as first-line treatment of metastatic non-small-cell lung cancer (GEMSTONE-302): interim and final analyses of a double-blind, randomised, phase 3 clinical trial (2022)	2	2	2	1	7
Sugemalimab versus placebo after concurrent or sequential chemoradiotherapy in patients with locally advanced, unresectable, stage III non-small-cell lung cancer in China (GEMSTONE-301): interim results of a randomised, double-blind, multicentre, phase 3 trial (2022)	2	2	2	1	7
Stereotactic body radiotherapy plus pembrolizumab and trametinib versus stereotactic body radiotherapy plus gemcitabine for locally recurrent pancreatic cancer after surgical resection: an open-label, randomised, controlled, phase 2 trial (2022)	2	2	0	1	5

Description: The score ranges from 0 to 7. Jadad score 0-3 indicates a low quality study, while higher scores (4-7) indicate high quality study.
